# FGFR4-associated APOBEC3 mutagenesis characterizes HER2-enriched subtype of human breast cancer

**DOI:** 10.1038/s41416-026-03517-8

**Published:** 2026-07-27

**Authors:** Ju-Yul Jeong, Chae-Yi Kim, A-Reum Nam, Je-Yoel Cho

**Affiliations:** 1 Department of Biochemistry, Brain Korea 21 Project and Research Institute for Veterinary Science, Seoul National University, College of Veterinary Medicine, Seoul, Republic of Korea; 2 Comparative Medicine Disease Research Center (CDRC), Science Research Center (SRC), Seoul National University, Seoul, Republic of Korea; 3 Present Address: Computational Cancer Genomics Group, Structural Biology Programme, Centro Nacional de Investigaciones Oncológicas (CNIO), Madrid, Spain

**Keywords:** Breast cancer, Cancer genomics

## Abstract

**Background:**

Characterising somatic mutation profiles in human breast cancer (HBC) is essential for understanding tumour progression and guiding therapeutic strategies.

**Methods:**

We performed genomic and transcriptomic analyses to profile the mutational landscape of HBC. In addition to our primary analysis of HBC, we conducted comparative genomic analyses to evaluate the extent to which these mutational processes are recapitulated in canine mammary tumour (CMT), a widely proposed translational model.

**Results:**

APOBEC3 (A3)-associated mutations were extensive in HBC but largely absent in CMT, likely due to structural differences in A3 proteins and lower basal expression. Transcriptomic stratification of HBC by A3 activity uncovered that tumours with A3 activity showed a strong association with the PAM50-HER2-Enriched (HER2E) subtype, and FGFR4 was significantly correlated with the expression and enzymatic function of APOBEC3A/B.

**Conclusions:**

These findings demonstrate that CMT is an unsuitable model for A3-mediated mutagenesis, emphasising the need to consider the limitations of cross-species mutational modelling in comparative oncology. Moreover, this study identifies a potential regulatory association between FGFR4 and A3, offering insights into the underlying mechanisms of A3-driven mutagenesis and suggesting that the FGFR4-A3 activity could be considered alongside other molecular biomarkers for the classification of the HER2E subtype.

## Introduction

Human breast cancer (HBC) continues to rise in incidence and has become the most commonly diagnosed cancer among women worldwide [1]. HBC is a biologically heterogeneous disease that develops through the interplay of multiple aetiological factors, including genetic predisposition, hormonal regulation, environmental exposures, and lifestyle-associated risks [2]. This biological diversity poses major challenges for effective prevention, diagnosis, and treatment, highlighting the necessity of elucidating the mechanisms that govern tumour initiation and progression.

Analysing somatic mutations through next-generation sequencing (NGS) has become a key approach to elucidating the molecular mechanisms of breast cancer development [3]. Diverse etiological factors lead to the progressive accumulation of somatic mutations, which drive tumour initiation, progression, and therapeutic resistance. Mutational signatures, defined as aggregate patterns of somatic alterations and their associated genomic activities, provide critical insights into these underlying processes by identifying endogenous biological activities and exogenous exposures implicated in tumorigenesis [4, 5]. Characterising these signatures not only enhances our understanding of the molecular drivers of HBC but also offers potential as predictive biomarkers for therapeutic responses.

Canine mammary tumours (CMTs) have been proposed as a potential translational model for HBC [6–8]. CMTs arise spontaneously in immunocompetent hosts and exhibit strong similarities to HBC in terms of clinicopathological features, histological subtypes, molecular classifications, and disease progression patterns. These shared features render CMTs a more physiologically relevant model for studying tumour biology, treatment response, and the tumour microenvironment than traditional rodent models. However, despite these similarities, it remains largely unknown whether CMTs recapitulate the complex genomic landscapes of HBC regarding cancer-associated mutational processes. Such uncertainty raises the possibility that CMTs may involve mutational mechanisms fundamentally distinct from those underlying the human disease, which would limit their validity as a translational model for certain mutagenic pathways.

In this study, we conducted a comprehensive analysis of somatic mutations in HBC to characterise mutational processes. In addition to the primary analyses of HBC, we performed comparative analyses of CMTs to evaluate how their genomic landscapes differ from those of HBC. Our findings reveal extensive APOBEC3 (A3)-associated mutagenesis in HBC, whereas CMTs exhibit limited mutational accumulation and A3 activity, providing a cautionary tale regarding the translational modelling of mutational mechanisms between these species. Moreover, we observed that A3 activity was strongly enriched in the HER2-Enriched (HER2E) subtype, as defined by the PAM50 classification. Functional analyses further demonstrated that the expression of FGFR4, which is one of the distinct features of this subtype, shows a significant positive correlation with the expression and enzymatic activity of APOBEC3A and APOBEC3B. Together, these findings identify a potential regulatory association between FGFR4 and A3-induced mutagenesis in HBC, suggesting that the FGFR4-associated A3 activation may represent a noteworthy molecular feature with clinical relevance.

## Results

### Mutational signature profiling of human breast cancer and canine mammary tumour enables comparative analysis

**Fig. 1:**
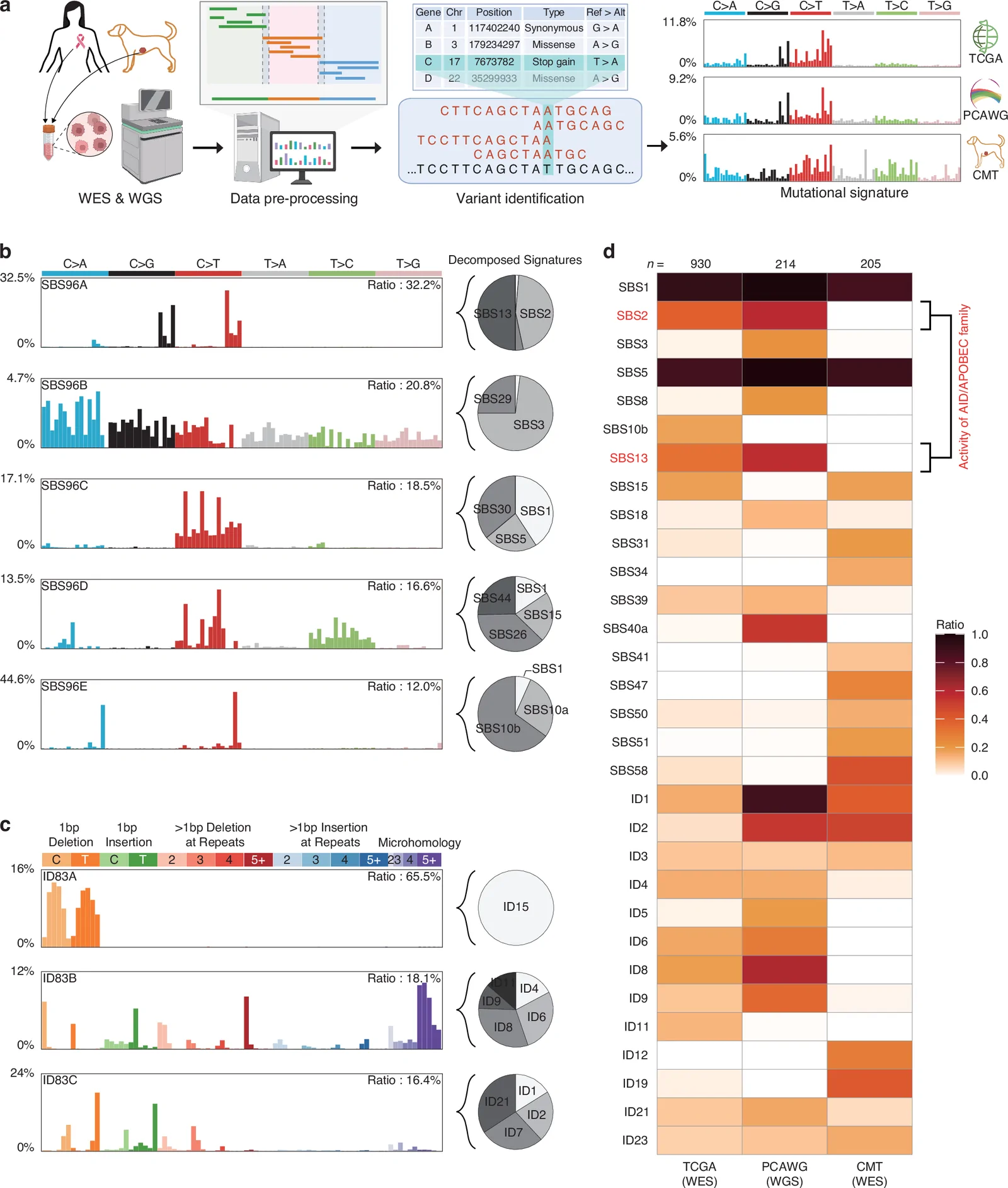
Comparative profiling of mutational signatures in human breast cancers and canine mammary tumours. **a** Overview of study design and analysis workflow. A total of 1349 samples were analysed, comprising human breast cancers from TCGA (WES, *n* = 930) and PCAWG (WGS, *n* = 214), along with CMTs (WES, *n* = 205). Partially created with BioRender.com (https://BioRender.com/iz41ar9). **b**, **c** De novo extraction of SBS and ID Mutational Signatures in HBC (TCGA). The ratio indicates the proportion of patients corresponding to each signature solution. For each solution, the proportions of human cancer signatures that constitute the solution are displayed as pie charts. **d** Comparison of signature prevalence across the TCGA, PCAWG, and CMT cohorts. Established human cancer signatures were directly assigned to mutational matrices; only those present in ≥10% of patients in at least one cohort and validated by corresponding de novo signatures were retained for visualisation. The ratio represents the proportion of patients harbouring each signature.

To investigate mutational processes in mammary tumours, we analysed sequencing datasets from both human and canine cohorts to characterise mutational signatures and species-specific features (Fig. 1a). For human breast cancer (HBC), whole-exome sequencing (WES, *n* = 930) and whole-genome sequencing (WGS, *n* = 214) datasets from The Cancer Genome Atlas (TCGA) and Pan-Cancer Analysis of Whole Genomes (PCAWG) project were analysed, respectively. In parallel, to serve as a comparative reference cohort for mutational processes in HBC, we analysed WES data from 205 paired canine mammary tumours (CMTs) and their matched adjacent normal tissues (Fig. 1a; Table S1) [9, 10]. Both tumour and normal samples exhibited high mapping quality and sufficient coverage depth for reliable variant detection (Table S2) [11–14]. Somatic mutations were identified by comparison of paired tumour and normal samples (Table S3) [15, 16]. These datasets were converted into mutation count matrices (SBS96 for single-base substitutions and ID83 for small insertions and deletions) for downstream mutational signature analysis [17].

Mutational signature analyses were conducted independently for three cohorts: TCGA, PCAWG, and CMT. For each cohort, de novo mutational signatures were extracted using a non-negative matrix factorisation (NMF) approach [18]. In the TCGA cohort, five SBS (SBS96A-E) and three ID signatures (ID83A-C) were identified as the optimal solution (Fig. 1b, c; Table S4). Among the extracted signatures, SBS2 and SBS13, associated with APOBEC3 (A3) cytidine deaminase activity, were detected in approximately one-third of TCGA samples, with a comparable prevalence observed in the PCAWG cohort, underscoring their substantial contribution to the mutational landscape of HBC (Table S4) [19].

In the CMT cohort, the optimal solution consisted of four SBS (SBS96A-D) and two ID (ID83A-B) signatures, none of which included A3-associated signatures (Fig. S1; Table S4). Most of the extracted CMT signatures showed high similarity to established human cancer signatures (cosine similarity >0.85), yet some appeared to resemble possible sequencing artefact (PSA) signatures [4]. Specifically, the ID83B signature exhibited a relatively low similarity (cosine similarity <0.70) alongside a high reconstruction error (Table S4). This discrepancy may be interpreted not as a technical failure, but rather as a “reference mismatch” inherent in applying human-derived models to canine data. Given that the COSMIC database is primarily curated from human mutational processes, it may lack the full explanatory power required for canine-specific profiles [20]. This suggests that the ID83B signature in CMTs represents a canine-specific mutational process distinct from the human processes archived in the COSMIC database, indicating a limitation where biological patterns unique to dogs can be misidentified as technical artefacts when interpreted solely through human-derived database.

Comparison of mutational processes across three cohorts (TCGA, PCAWG, and CMT) identified cohort-specific signatures (Fig. 1d; Table S5). Notably, SBS2 and SBS13, which are attributed to A3-mediated cytidine deamination, were detected in 39% and 33% of TCGA samples and in 59% and 57% of PCAWG samples, respectively, but were not detected in the CMT cohort (0%). A3, a potent driver of mutagenesis in human cancers, contributes substantially to the overall mutational burden, with a reported prevalence in approximately 50% of all cancer genomes and in over 70% of cancer types [21–23]. This finding suggests that A3-driven mutagenesis may be a mutational process specific to human breast cancer, and its absence in CMT underscores a fundamental genomic divergence that warrants further investigation into its underlying molecular causes.

### APOBEC3-driven mutational burden was detected in HBC but absent in CMT

Among the diverse mutational processes associated with cancer, A3-mediated mutagenesis has been recognised as a prominent endogenous source of somatic mutations in multiple tumour types, including HBC, contributing to genomic instability, tumour heterogeneity, and poor clinical outcomes [21–25]. Since the activation status of A3 can serve as a fundamental driver that defines the genomic landscape of a tumour, accurately identifying its activity is of paramount importance. However, given its strong mutagenic potential, it remains unclear whether A3 activity is consistently represented in the WES-derived mutational landscapes of HBC, and whether the absence of A3-associated signatures observed in the CMT cohort is likewise reflected at the whole-exome level.

To delineate the activation status of A3 through a conservative approach and to quantify the extent of A3-associated mutagenesis, WES data from 930 TCGA HBC samples were stratified into two groups: those harbouring at least one A3-associated signature (SBS2 and/or SBS13) (A3 + , *n* = 402) and those lacking both signatures (A3-, *n* = 528). All 205 CMT samples were included for parallel comparison. For each of the three groups, single-base substitution (SBS) mutations were extracted from coding regions and classified as total, missense, or synonymous. These categories were then compared across groups to assess differences in mutational distributions and to evaluate the impact of A3 activity on mutational accumulation.

**Fig. 2:**
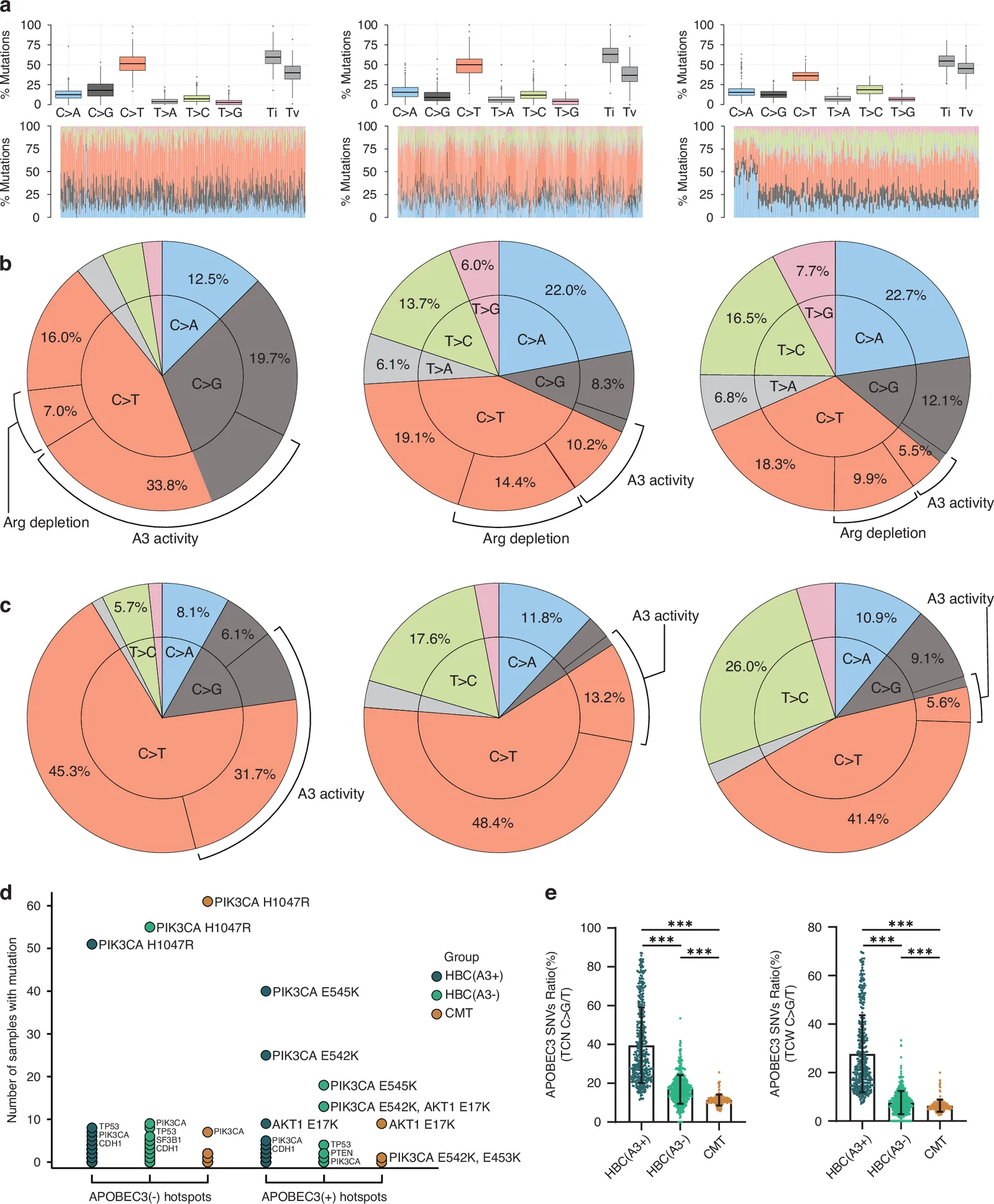
Differential APOBEC3-driven mutagenesis between human breast cancers and canine mammary tumours. **a** Distribution of coding sequence mutations in HBC(A3 + ) (left, *n* = 374), HBC(A3-) (centre, *n* = 496), and CMT (right, *n* = 205). Mutations are classified into six categories: C > A (blue), C > G (black), C > T (red), T > A (grey), T > C (olive), and T > G (pink). Ti denotes transition mutations, and Tv denotes transversion mutations. The lower panel shows the proportion of mutation classes in individual patients. **b** Distribution of missense mutations across the three groups, as in (**a**). Mutations associated with APOBEC3 (A3) activity and with arginine (Arg) depletion-related regions were categorised separately. For missense mutations associated with A3 activity, 95% CIs of mean diff. and effect sizes (Cohen’s d) are as follows: HBC(A3 + ) (mean of ratios = 24.54%) vs. HBC(A3-) (mean of ratios = 10.84%) (mean diff. 12.19–15.21%, d = 1.32). CMT (mean of ratios = 5.60%) vs. HBC(A3-) (mean of ratios = 10.84%) (mean diff. −6.14 to −4.34%, *d* = −0.80). CMT (mean of ratios = 5.60%) vs. HBC(A3 + ) (mean of ratios = 24.54%) (mean diff. −20.44 to −17.43%, d = −1.70). **c** Distribution of synonymous mutations across the three groups, as in (**a**). Mutations associated with A3 activity were categorised separately. For synonymous mutations associated with A3 activity, 95% CIs of mean diff. and effect sizes (Cohen’s d) are as follows: HBC(A3 + ) (mean of ratios = 23.27%) vs. HBC(A3-) (mean of ratios = 11.26%) (mean diff. 9.91–14.11%, d = 0.79). CMT (mean of ratios = 5.14%) vs. HBC(A3-) (mean of ratios = 11.26%) (mean diff. −7.60 to −4.64%, d = −0.49). CMT (mean of ratios = 5.14%) vs. HBC(A3 + ) (mean of ratios = 23.27%) (mean diff. −19.97 to −16.28%, d = −1.33). **d** Distribution of oncogene hotspot mutations across the three groups, as in (a): HBC(A3 + ) (dark cyan), HBC(A3-) (vivid green), and CMT (vivid orange). Oncogene hotspot mutations were classified as A3(+) hotspots when located within A3-associated regions, and as A3(−) hotspots when not associated with A3, and are listed accordingly. **e** Proportions of C > T or C > G substitutions within A3-preferred sequence contexts (5′-TCN-3′ and 5′-TCW-3′) across three groups: HBC(A3 + ) (dark cyan, *n* = 402), HBC(A3-) (vivid green, *n* = 528), and CMT (vivid orange, *n* = 205). In the TCN context, N denotes any base, whereas in the TCW context, W corresponds to either A or T. The proportion was calculated by dividing the number of mutations within A3-preferred contexts by the total number of mutations per sample. Statistical significance was determined using the two-sided Brown-Forsythe and Welch’s ANOVA followed by Games-Howell’s multiple comparison test. Data are presented as means ± SD. For TCN context, 95% CIs of mean diff. and effect sizes (Cohen’s d) are as follows: HBC(A3 + ) vs. HBC(A3-) (20.29-25.09%, d = 2.01), CMT vs. HBC(A3-) (-6.34 to -4.57%, d = 1.15), and CMT vs. HBC(A3 + ) (-30.47 to -25.82%, d = 2.14). For TCW context, HBC(A3 + ) vs. HBC(A3-) (18.14-22.00%, d = 1.14), CMT vs. HBC(A3-) (0.70-1.98%, d = 0.19), and CMT vs. HBC(A3 + ) (19.49-23.31%, d = 1.26). ***P < 0.001.

The overall mutational landscape, including the proportions of transitions and transversions, was examined (Fig. 2a) [26]. The total number of mutations was 38,591 in HBC(A3 + ), 32,848 in HBC(A3-), and 17,429 in CMT. In both HBC groups, C > T substitutions were the most frequent, accounting for more than half of all mutations, likely reflecting spontaneous deamination of 5-methylcytosine at CpG sites [27]. In HBC(A3 + ), C > G substitutions were the second most common, whereas T > A, T > C, and T > G substitutions occurred at low frequencies. This distribution indicates that in the presence of A3 activity, its characteristic signature dominates the mutational spectrum, thereby diminishing the relative contributions of other mutagenic processes. In CMT, C > T substitutions were also the most prevalent, but T > C ranked second, and the relative proportions of T > A and T > G were higher than in HBC, suggesting a distinct pattern of mutational accumulation in CMT compared with HBC.

Missense mutations totalled 28,444 in HBC(A3 + ), 24,643 in HBC(A3-), and 8,777 in CMT (Fig. 2b). Variants typically associated with A3 activity were observed, including E > K, E > Q, S > L, S > F, S > C, D > N, D > H, and Q>Ter substitutions [21, 28, 29]. Mutations within arginine-depleted regions, represented by R > Q, R > C, R > H, and R > W substitutions, were also catalogued, as these have been reported to be enriched under specific tumour microenvironmental conditions [30–33]. The HBC(A3 + ) group exhibited a higher missense mutation load per sample than HBC(A3-), consistent with the contribution of A3 activity, with A3-associated variants accounting for 33.8% of the pooled missense burden. In contrast, HBC(A3-) and CMT displayed broadly similar distributions of missense classes, with A3-related variants comprising only 10.2% and 5.5% of their respective pooled missense mutations, respectively. Arginine depletion mutations represented 7% of all missense variants in HBC(A3 + ), a proportion lower than that observed in HBC(A3-) and CMT.

Synonymous mutations were also analysed, totalling 9531 in HBC(A3 + ), 7575 in HBC(A3-), and 6226 in CMT (Fig. 2c). Among these, substitutions characteristic of A3 activity, such as NTC > NTT and NTC > NTG (where N represents any base), were identified. Similar to the missense mutation patterns, A3-associated substitutions accounted for 31.7% of all synonymous mutations in HBC(A3 + ), whereas their proportions in HBC(A3-) and CMT were substantially lower and showed comparable distributions. Collectively, these findings indicate that A3 activity is a major determinant of coding sequence mutational accumulation in HBC, while CMT lacks significant A3-associated mutagenesis and exhibits a profile that relatively resembles that of HBC(A3-).

To further delineate the impact of A3 activity, its contribution to oncogene hotspot mutations was evaluated (Fig. 2d; Table S6). Statistically significant hotspot mutations were compiled from large-scale cancer genomics datasets and classified into A3(+) hotspots (*n* = 42), consistent with A3 mutagenesis, and A3(-) hotspots (*n* = 215), unrelated to A3 activity [34–36]. Comparative analysis among HBC(A3 + ), HBC(A3-), and CMT revealed that the A3-associated PIK3CA hotspots E545K and E542K were most frequent in HBC(A3 + ) (*n* = 40 and 25, respectively), approximately twice the counts observed in HBC(A3-) (*n* = 18 and 13, respectively). In CMT, mutations corresponding to A3(+) hotspots were largely absent, and E545K and E542K also occurred only at very low frequencies. In contrast, analysis of A3(-) hotspots showed that the PIK3CA H1047R mutation was the most prevalent in all three groups (*n* = 51 in HBC(A3 + ), *n* = 55 in HBC(A3-), and *n* = 61 in CMT), whereas other A3(−) hotspots occurred at uniformly low frequencies across groups.

Mutation frequencies within the A3-preferred sequence contexts 5′-TCN-3′ and 5′-TCW-3′ (where N is any base and W is A or T) were compared across the three groups (Fig. 2e) [21, 23, 37, 38]. The SBS96 mutation count matrices for each group were used to quantify C > T or C > G substitutions occurring in A3-associated contexts. In HBC(A3 + ), mutations in the TCN and TCW contexts accounted for 11.54-87.09% and 7.14–69.78% of all mutations, with mean proportions of 39.53% and 27.72%, respectively. In contrast, mean proportions in HBC(A3-) were 16.84% (TCN) and 7.66% (TCW), and CMT showed values of 11.38% and 6.32%, respectively. These results, consistent with the earlier mutational signature analysis, further confirm that A3 activity contributes substantially to mutational accumulation in HBC while remaining negligible in CMT, highlighting a divergence in the endogenous mutagenic landscape between the two species.

### Canine APOBEC3 orthologs exhibit low sequence but high structural similarity to the human APOBEC3 family

Comparative analysis of mutation data from HBC and CMT indicated that mutation accumulation driven by A3 activity was limited in CMT. In dogs, the orthologs corresponding to the human A3 gene family (APOBEC3A-H) are APOBEC3Z1 (A3Z1) and APOBEC3Z3 (A3Z3) [39, 40]. To explore potential molecular explanations for the restricted A3 activity observed in CMT, the sequence and structural characteristics of canine A3Z1 and A3Z3 were analysed in comparison with their human A3 counterparts.

For comparison of A3 genes between humans and dogs, both coding and amino acid sequences were analysed (Fig. S2a). Pairwise BLAST comparisons of human APOBEC3A-H (A3A-H) and the canine orthologs A3Z1 and A3Z3 revealed no significant nucleotide sequence similarity in any pair. Consistently, amino acid sequences showed low similarity, with sequence identity values below 50% across all comparisons.

In contrast, structural comparison revealed high similarity between human and canine A3 proteins. Using the TM-align algorithm, we evaluated the predicted three-dimensional structures of each protein pair by calculating the Root Mean Square Deviation (RMSD) and Template Modelling Score (TM-score) [41]. Most pairs between human A3A-H and canine A3Z1 or A3Z3 showed significant structural similarity (RMSD < 2 Å and TM-score > 0.5) (Fig. S2b). In particular, several pairs involving A3Z1 (with A3A, A3B, and A3G) and A3Z3 (with A3H) exhibited the highest similarity, with TM-scores ≥0.9. Control comparisons showed low similarity for TP53 and CDH1, whereas PIK3CA and PTEN displayed high structural similarity. These findings indicate that while canine A3 orthologs exhibit low sequence identity with the human A3 family, they maintain a conserved structural scaffold. This high degree of structural similarity suggests that the basal catalytic mechanisms, such as the deaminase activity common to the A3 family, are likely preserved in canine orthologs [21, 40, 42]. However, whether this conserved architecture extends to the capacity for driving somatic mutations in the cancer genome remains to be determined.

### Loop region divergence underlies functional decoupling of structurally conserved APOBEC3 orthologs

To explain the functional differences of A3 in between human and canine, the four pairs exhibiting the highest structural similarity (A3A with A3Z1, A3B with A3Z1, A3G with A3Z1, and A3H with A3Z3) were subjected to detailed structural analysis. Amino acid sequences for each pair were aligned, and secondary structural features were visualised based on the alignments (Fig. S3a–d) [43, 44]. Across all pairs, α-helices and β-sheets were positioned at comparable locations, whereas major sequence differences were predominantly observed in the loop regions connecting secondary structural elements.

Previous studies have identified A3A, A3B, A3G, and A3H as the major A3 family members involved in mutagenesis in breast cancer [21, 34, 45, 46]. Among them, A3A exhibits the strongest cytidine deaminase activity despite its transient expression, whereas A3B is less active enzymatically but broadly expressed, both of which contributes substantially to mutagenesis. The enzymatic activity of A3s is primarily governed by the sequences of loop regions 1, 3, and 7, which influence substrate access and catalytic efficiency. As a result, individual A3 proteins exhibit distinct substrate preferences, determined by substrate structure, hairpin loop length, and the active site context.

**Fig. 3:**
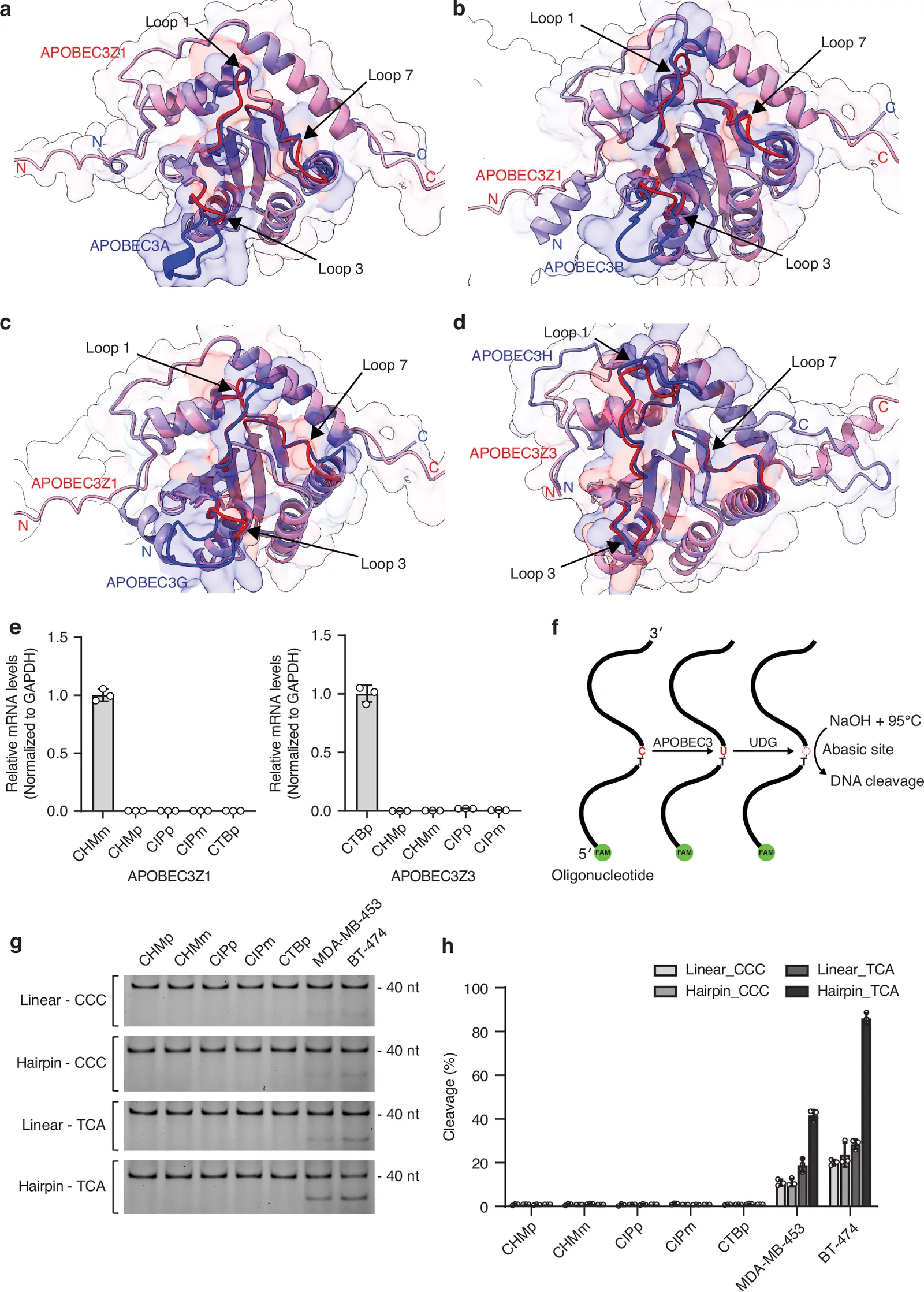
Comparative analysis of APOBEC3 orthologs and restricted activity in canine mammary tumours. **a**–**d** Molecular surfaces of canine APOBEC3Z1 and APOBEC3Z3 aligned with human APOBEC3 (A3) proteins. Canine APOBEC3Z1 (UniProt ID: A0A8I3PIE8) and APOBEC3Z3 (UniProt ID: A0A7R6SZJ1) are shown in red, while their human counterparts APOBEC3A (UniProt ID: P31941), APOBEC3B (UniProt ID: Q9UH17), APOBEC3G (UniProt ID: Q9HC16), and APOBEC3H (UniProt ID: Q6NTF7) are shown in blue. Loop regions 1, 3, and 7 are shown in darker shades to highlight structural elements. The N-terminus and C-terminus of each protein are labelled as N and C, respectively. Protein structures were retrieved from UniProt IDs and corresponding PDB files, pairwise sequence alignments were performed using the Needleman-Wunsch algorithm, and molecular surfaces were visualised with ChimeraX. **e** Relative mRNA expression levels of A3Z1 and A3Z3 in five CMT cell lines (CHMp, CHMm, CIPp, CIPm and CTBp), measured by RT-qPCR. P-values are not explicitly indicated for visual clarity. **f** Schematic representation of the in vitro APOBEC3 deaminase activity assay. In this assay, cytosine residues within specific sequence contexts are deaminated to uracil by A3, excised by uracil DNA glycosylase (UDG), and converted into abasic sites (AP sites). Under alkaline conditions at 95 °C, these AP sites become unstable, resulting in DNA cleavage. **g** A3 activity in the indicated CMT and HBC cell lines, measured using linear (CCC or TCA) or hairpin (CCC or TCA) substrates. The added oligonucleotides were 40 nt in length and yielded 21 nt products upon cleavage by A3. **h** Quantification of A3 activity, expressed as the percentage of cleaved DNA from panel (**g**), calculated as the intensity of the 21 nt band relative to the 40 nt band, and quantified using ImageJ. The substrates used in the experiments were Linear-CCC (light grey), Hairpin-CCC (grey), Linear-TCA (dark grey), and Hairpin-TCA (charcoal). *P*-values are not explicitly indicated for visual clarity.

To further investigate differences in loop structures between human and canine A3 proteins, comparative analyses of their predicted three-dimensional structures were performed. The four pairs with the highest structural similarity were modelled (Fig. 3a–d) [47]. Overall, α-helices and β-sheets were consistently aligned across all pairs, although structural divergence was apparent in loops 1, 3, and 7. Notably, loop 7 showed pronounced divergence, which is significant given its role in recognising the TpC motif, the primary target sequence associated with oncogenic mutational signatures [48, 49]. The reliability of these structural comparisons was validated through predicted Local Distance Difference Test (pLDDT) scores and Predicted Aligned Error (PAE) analysis of loops 1, 3, and 7 (Fig. S4a–g). Although localised residue-level uncertainty was observed in specific segments, the overall structural predictions for the key functional loops remained within a reliable confidence range, supporting the robustness of the comparative assessment.

In summary, while human and canine A3 orthologs exhibit α-helices and β-sheets aligned in similar positions, this global similarity does not fully account for the functional nuances driven by loop sequences. The enzymatic activity of A3 proteins is strictly governed by loop composition, where sequence divergence can alter loop conformation, restrict substrate access to the active site, and dictate substrate specificity. These findings collectively suggest that the divergence in loop sequences directly influences catalytic activity. Given that canine A3 orthologs possess loop sequences distinct from the human A3 members typically responsible for oncogenic mutagenesis, this structural disparity likely explains why A3-related mutational signatures were not detected in CMT. These results further suggest that the mutational burden exerted by A3 is either minimal in dogs or that canine A3 proteins target different motifs than those observed in humans.

### Functional validation reveals absence of APOBEC3 activity in CMT cells

Structural analyses indicated that canine A3 proteins contain loop sequences distinct from their human counterparts, potentially affecting substrate preference and enzymatic activity. To assess these functional implications, we evaluated A3 expression and function in canine mammary tumour (CMT) cell lines (CHMp, CHMm, CIPp, CIPm, and CTBp), along with potential differences in substrate specificity relative to human A3 proteins [50–52].

Expression examination across five CMT cell lines revealed that A3Z1 was detected only in CHMm (average ΔCq = 10.32), whereas A3Z3 was expressed in all cell lines, with the highest level observed in CTBp (average ΔCq = 8.24) (Fig. 3e; Fig. S5) [53]. This suggests that canine A3 orthologs are expressed at low basal levels, and that the restricted expression of A3Z1, despite its high structural similarity to its human counterpart, may influence the overall activity of A3 proteins.

To evaluate the mutagenic potential of A3Z1 and A3Z3, an in vitro APOBEC3 deaminase activity assay was performed (Fig. 3f). Previous studies have shown that human A3A and A3B preferentially act on hairpin oligonucleotides, while A3G and A3H prefer linear oligonucleotides, with A3G exhibiting specificity for the 5′-CCC-3′ context [23, 34, 54, 55]. Based on these preferences, four substrate types were tested: Linear-CCC, Hairpin-CCC, Linear-TCA, and Hairpin-TCA (Fig. S6). MDA-MB-453 and BT-474 HBC cell lines, which have documented A3 activity, served as positive controls [23]. No DNA cleavage was detected in any of the five CMT cell lines under any substrate condition, whereas both HBC cell lines showed clear evidence of cleavage (Fig. 3g, h) [56].

Together, these findings demonstrate that CMT cells lack detectable A3 activity, resulting in a restricted A3-assoicated mutational accumulation compared with human breast cancer. This absence of activity is likely attributable to both structural divergence in loop sequences that are critical for substrate recognition and catalysis, and to intrinsically low basal expression. These results reveal species-specific divergences in CMT as a mutational modelling system, suggesting that caution should be exercised when extrapolating canine mutational data to human cancer biology.

### Transcriptomic profiling links APOBEC3 activity to the HER2-Enriched subtype in breast cancer

**Fig. 4:**
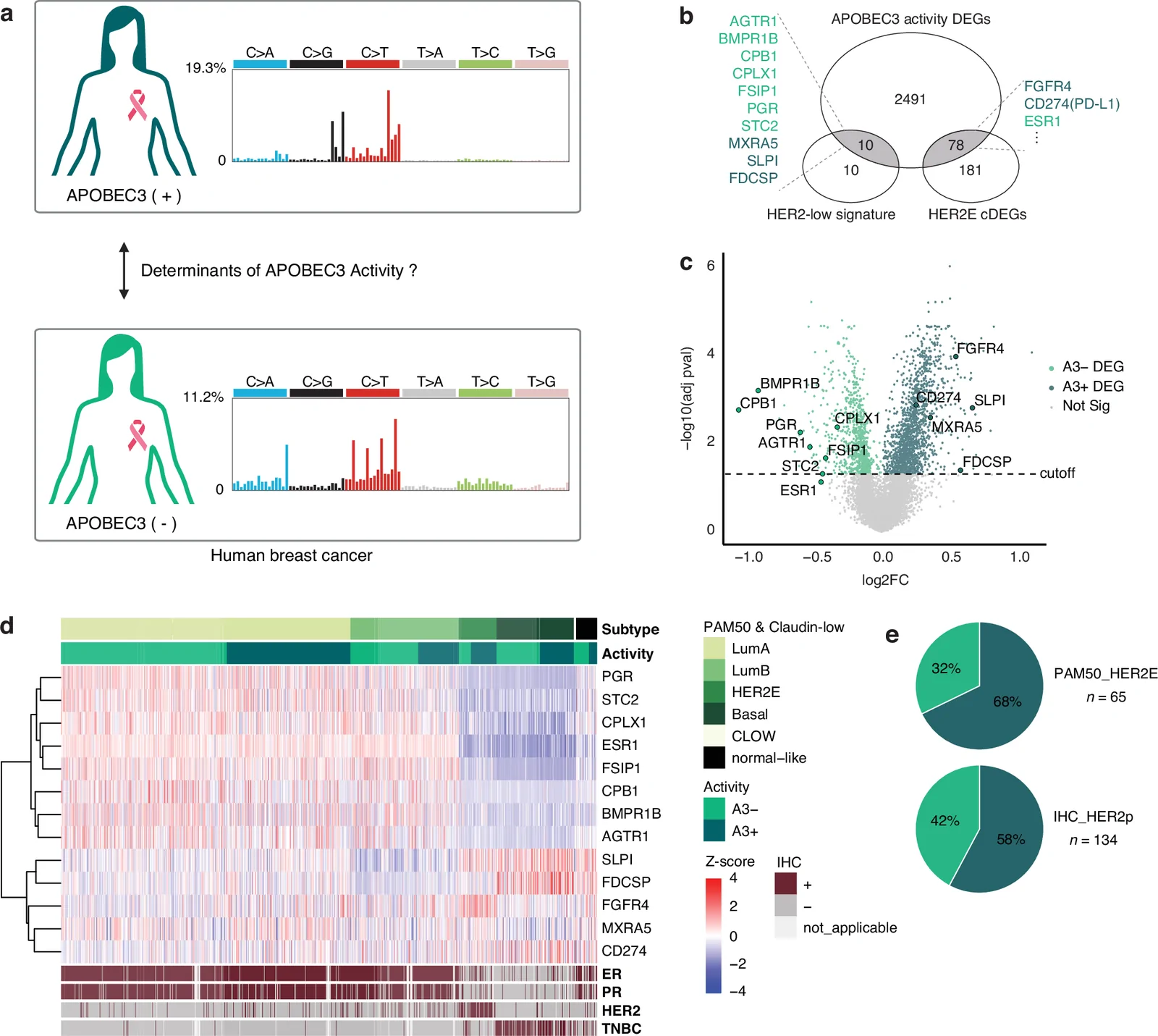
Elevated APOBEC3 Activity in PAM50-HER2-Enriched Breast Cancers. **a** Graphical scheme highlighting the determinants contributing to APOBEC3 (A3) activity. Partially created with BioRender.com (https://BioRender.com/iz41ar9). **b** Venn diagram and (**c**). Volcano plot of differentially expressed genes (DEGs) identified in TCGA-BRCA patients, stratified by the presence or absence of A3 activity: A3+ (*n* = 401) and A3- (*n* = 526). Representative HER2 subtype-associated DEGs reported in previous studies are visualised. Genes highlighted in dark cyan represent DEGs enriched in the A3+ group, while those in vivid green represent DEGs in the A3- group. The significance threshold was set at adj. *p* < 0.05; Notably, STC2 (adj. *p* = 0.0502) and ESR1 (adj. *p* = 0.0806) are also indicated on the plots. **d** Heatmap of RNA-seq expression levels in TCGA-BRCA patients (*n* = 927) based on representative HER2 subtype-associated DEGs. For each patient, histologic subtype information (PAM50 and IHC) reported in previous studies, along with A3 activity status (SBS2/SBS13 signatures), is visualised. Expression values were scaled via Z-score normalisation across samples. **e** Proportion of cases with A3 activity across IHC-HER2 or PAM50-HER2E subtypes.

HBC exhibited pronounced differences in mutation burden depending on the presence or absence of A3 activity. However, the definitive inducers and consistent patient characteristics associated with elevated A3 activity remain incompletely understood [21–23]. Addressing these gaps is essential for delineating patient-specific mutational programmes and their potential therapeutic relevance. Given the potential of A3 as a therapeutic target, it is critical to identify key factors associated with A3 activity in HBC (Fig. 4a).

To investigate whether A3 activity arises from gene expression or from interactions with additional regulatory factors, RNA-seq data from TCGA-BRCA were analysed. Expression levels of A3A, A3B, A3G, and A3H, which are implicated in A3 mutagenesis in breast cancer, were compared between the HBC(A3 + ) and HBC(A3-) groups (Fig. S7a–d) [21]. Consistent with previous reports, A3A showed low basal expression in both groups, whereas A3B was highly expressed in tumour samples relative to matched normal tissue samples [21, 45, 46]. Across all four genes, expression levels were significantly higher in the A3+ tumours compared with the A3- tumours. Moreover, even in the absence of detectable A3 activity, the A3- tumours generally exhibited higher A3 expression than the corresponding normal tissues. These findings indicate that A3 activity is not determined solely by expression levels but is likely regulated through interactions with additional molecular determinants.

To identify key molecular factors associated with A3 activity, differential gene expression (DEG) analysis was conducted between the A3+ and A3- groups. Examination of the DEG lists revealed that many genes were associated with the HER2 molecular subtype of HBC (Fig. 4b, c; Table S8). Among the DEGs significantly upregulated in the A3+ group (MXRA5, SLPI, and FDCSP) and those in the A3- group (AGTR1, BMPR1B, CPB1, CPLX1, FSIP1, PGR, and STC2), all were included in a 20-gene signature reported to distinguish HER2-low primary breast tumours [57]. In principal component analysis of this signature, A3+ group DEGs showed negative coefficients, whereas A3- group DEGs showed positive coefficients. This pattern indicates that the A3- group displays a transcriptomic profile more closely resembling the HER2-low phenotype compared with the A3+ group. Considering that HER2-low tumours share genomic and clinical features with HER2-negative breast cancer, these findings imply that the A3+ group is more likely to exhibit a HER2-positive (HER2p) phenotype [58–60].

In addition, 78 DEGs, including FGFR4 and CD274 (PD-L1) from the A3+ group and ESR1 from the A3- group, have been reported as core DEGs (cDEGs) for the HER2-Enriched (HER2E) subtype [61]. The HER2E subtype represents a heterogeneous entity that encompasses diverse molecular phenotypes. While it is primarily characterised by ERBB2 overexpression, this subtype also includes a distinct patient subset more closely aligned with FGFR4 signalling. Although ERBB2/GRB7 are recognised as major contributors to the PAM50 definition of HER2E, FGFR4 overexpression can exert a more predominant influence in specific clinical subgroups. CD274, although not included in the HER2E cDEG set, is expressed at higher levels in HER2E tumours compared with LumA or LumB subtypes and serves as a biomarker of immune cell infiltration within the tumour microenvironment. Collectively, these observations indicate that the A3+ group exhibits molecular features consistent with the FGFR4-high HER2E phenotype. This suggests a potential functional association between A3 activity and the specific signalling environment characteristic of the HER2E subtype.

Expression patterns of 13 DEGs in A3+ and A3- patient groups were further visualised by histologic type using a heatmap (Fig. 4d) [62]. Genes included in the HER2-low signature showed marked differential expression predominantly in hormone receptor-positive PAM50-LumA and LumB subtypes [58–60]. Comparison of PAM50 subtyping with IHC results revealed instances in which IHC-HER2p patients were classified as PAM50-LumA and IHC-HER2-negative patients were classified as PAM50-HER2E. These findings highlight that IHC-based classification alone may be insufficient for accurate identification of HER2E patients. A3 activity was detected across all PAM50 subtypes, with the highest levels observed in HER2. This activity was more frequent in PAM50-HER2E (68%) than in IHC-HER2p cases (58%) (Fig. 4e). To assess whether the observed association between the HER2E subtype and A3 activity was statistically significant, we evaluated its distribution across PAM50 subtypes (Table 1). A global χ² test confirmed that A3 activity was not evenly distributed among subtypes (χ² = 21.85, df = 5, *p* = 5.6 × 10⁻⁴). Among them, the HER2E group showed a significantly higher prevalence of A3 activity compared with all other subtypes, with an odds ratio of 2.96 and a relative risk of 1.63, corresponding to an absolute risk difference of 26 percentage points. None of the other PAM50 subtypes exhibited significant associations after multiple-testing correction.

**Table 1:** Statistical association between PAM50 subtypes and APOBEC3 activity in breast cancer.

Subtype	A3+ cases	A3- cases	OR (95% CI)	Fisher’s exact P-value	RR	RD (%)	Adjusted p (Holm)
LumA	213	286	0.95 (0.72–1.24)	0.69	0.97	−1.3	1
LumB	70	117	0.74 (0.52–1.04)	0.083	0.84	−7.3	0.41
HER2-Enriched	44	21	2.96 (1.69–5.33)	4.6 × 10⁻⁵	1.63	26	2.8 × 10⁻⁴
Basal	59	74	1.05 (0.71–1.55)	0.85	1.03	1.3	1
Normal-like	14	22	0.83 (0.39–1.72)	0.61	0.89	−4.6	1
CLOW	0	4	0.0 (0.00–1.98)	0.138	0	−43.5	0.55

To investigate whether APOBEC3 (A3) activity is differentially distributed across breast cancer molecular subtypes, we first performed an overall association test using the Chi-square test and Fisher’s exact test (with 10^6^ Monte Carlo simulations). For subtype-specific associations, each subtype was compared against all other subtypes combined using two-sided Fisher’s exact tests based on 2 × 2 contingency tables. To quantify the magnitude of association, odds ratios (ORs) with 95% confidence intervals (CIs), relative risks (RRs), and risk differences (RDs) were calculated. To control for the family-wise error rate in multiple subgroup comparisons, P-values were adjusted using the Holm-Bonferroni method (Holm’s step-down procedure). An adjusted *P*-value < 0.05 was considered statistically significant. All statistical analyses were performed using R with the epitools and binom packages.

In summary, differential expression analysis to identify key molecular factors associated with A3 activity in HBC revealed that this activity is detectable across all molecular subtypes but is pronounced in the HER2 subtype, with the highest levels observed in the PAM50-HER2E group. Unlike ERBB2 overexpression, which is specific to the HER2E subtype, FGFR4 overexpression can also be detected in other molecular contexts, including Luminal A and B subtypes [61, 63]. This distinct distribution pattern may explain why A3 activity is observed in certain non-HER2E cases or conversely, why it is absent in a subset of HER2E patients who lack sufficient FGFR4 expression. Together, these results demonstrate a functional and statistical association between A3 activity and the PAM50-HER2E molecular subtype, suggesting that FGFR4 overexpression, as one of the hallmark features of this phenotype, likely plays a role in modulating A3 activity.

### Screening of breast cancer cell lines suggests that the observed activity tends to align with APOBEC3B expression

The HER2E PAM50 molecular subtype displayed higher A3 activity than other subtypes, suggesting potential functional interactions with specific gene sets. To explore these interactions, PAM50-based subtyping analysis was conducted for a panel of breast cancer cell lines (Fig. S8; Table S9). Notably, MCF-7 and T-47D, traditionally classified as LumA, exhibited greater transcriptional similarity to the LumB subtype, while MDA-MB-231, typically assigned to the Basal subtype, showed partial transcriptional overlap with LumB.

**Fig. 5:**
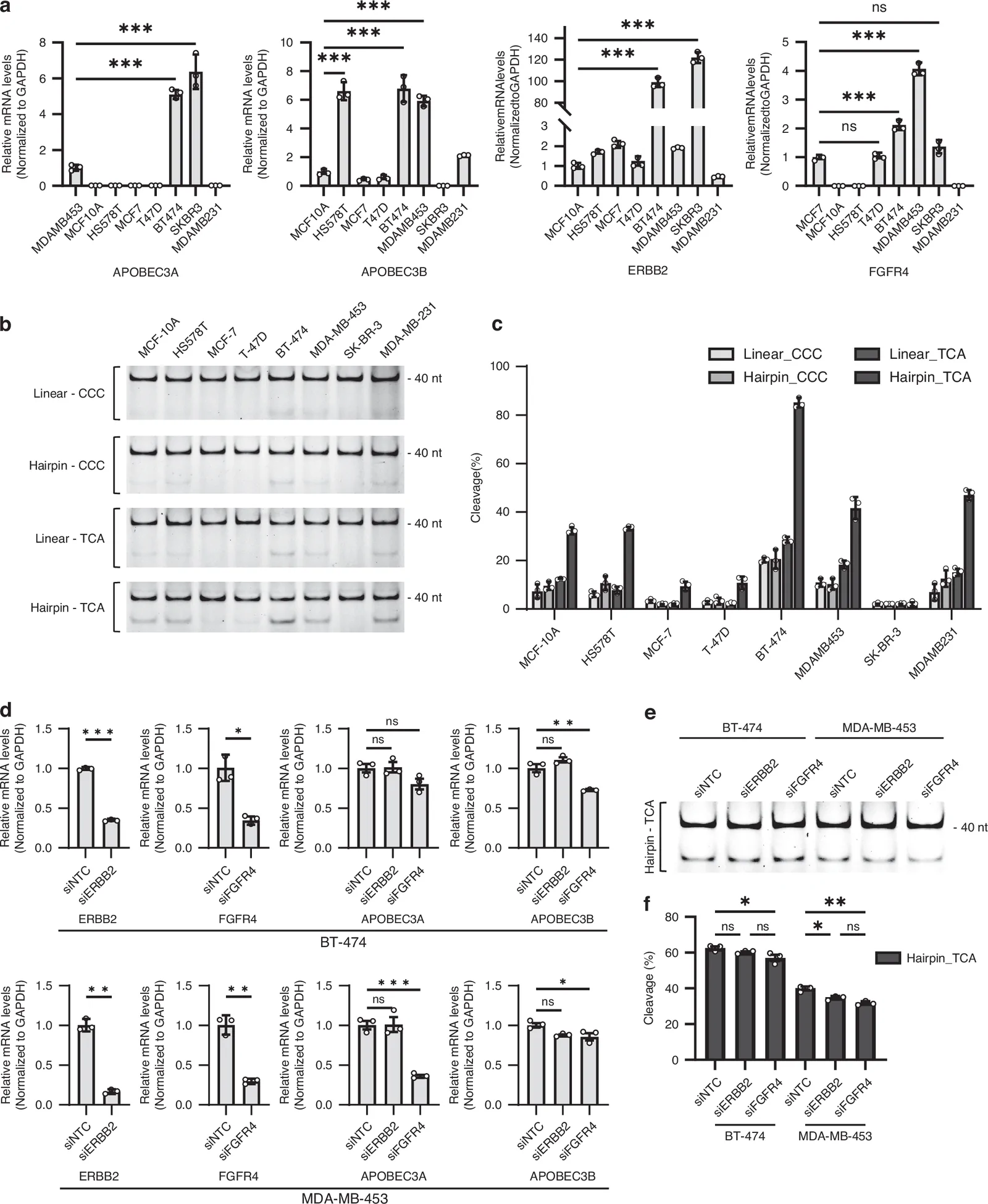
FGFR4 contributes to APOBEC3 activity in breast cancer cell lines. **a** Relative mRNA expression levels of APOBEC3A, APOBEC3B, ERBB2, and FGFR4 in eight HBC cell lines (MCF-10A, HS-578T, MCF-7, T-47D, BT-474, MDA-MB-453, SK-BR-3 and MDA-MB-231), measured by RT-qPCR. ***P < 0.001; ns, not significant. **b** APOBEC3 (A3) activity in the indicated HBC cell lines, measured using linear (CCC or TCA) or hairpin (CCC or TCA) substrates. The added oligonucleotides were 40 nt in length and yielded 21 nt products upon cleavage by A3. **c** Quantification of A3 activity, expressed as the percentage of cleaved DNA from panel B, calculated as the intensity of the 21 nt band relative to the 40 nt band, and quantified using ImageJ. The substrates used in the experiments were Linear-CCC (light grey), Hairpin-CCC (grey), Linear-TCA (dark grey), and Hairpin-TCA (charcoal). P-values are not explicitly indicated for visual clarity. **d** Relative mRNA expression levels of ERBB2, FGFR4, APOBEC3A, and APOBEC3B following siRNA knockdown of non-targeting control (siNTC), ERBB2 (siERBB2), or FGFR4 (siFGFR4) in BT-474 and MDA-MB-453 cell lines. *P < 0.05, **P < 0.01, ***P < 0.001; ns, not significant. For APOBEC3B mRNA expression in MDA-MB-453 cells, both siNTC-siFGFR4 and siNTC-siERBB2 comparisons showed similar mean differences (0.146 and 0.126, respectively). However, only the siFGFR4 comparison reached statistical significance, as its 95% confidence interval did not include zero (0.0077 ~ 0.283). In contrast, the interval for siERBB2 included zero (-0.0118 ~ 0.264). **e** A3 activity in BT-474 and MDA-MB-453 cells following siRNA knockdown of siNTC, siERBB2, or siFGFR4. **f** Quantification of A3 activity from panel (**e**), expressed as the percentage of cleaved DNA. Data are presented as means ± SEM. *P < 0.05, **P < 0.01; ns not significant.

Given the potential association between A3 activity and HER2E biomarkers such as ERBB2 and FGFR4, we quantified mRNA expression levels of ERBB2, FGFR4, A3A, and A3B, across a panel of eight breast cancer cell lines (Fig. 5a; Fig. S9) [61]. A3A expression was detected exclusively in the HER2E cell lines MDA-MB-453 and SK-BR-3, as well as in the LumB cell line BT-474. In contrast, A3B was expressed in all cell lines except SK-BR-3. ERBB2 expression was highest in SK-BR-3, followed by BT-474 and MDA-MB-453, whereas FGFR4 expression was highest in MDA-MB-453, followed by BT-474 and SK-BR-3.

To evaluate mutagenic potential, an in vitro APOBEC3 deaminase activity assay was performed (Fig. 5b, c). DNA cleavage was most pronounced with the hairpin-TCA substrate, which is preferred by A3A and A3B. Across cell lines, cleavage levels were generally higher with the linear-TCA substrate than with hairpin-CCC, indicating that sequence context (TCA) exerted a greater influence on A3 activity than substrate structure (linear or hairpin). Among the tested lines, BT-474 (LumB) showed the strongest activity, whereas SK-BR-3 (HER2E) exhibited no detectable activity. Considering the mRNA expression profiles of A3A and A3B, the observed activity is most consistent with A3B function, with minimal or no contribution from A3A.

### FGFR4 knockdown reduces APOBEC3 expression and activity in HER2E-like breast cancer cell line

Evaluation of A3 expression and activity in breast cancer cell lines did not allow a definitive interpretation of the effects of the HER2E biomarkers ERBB2 and FGFR4 on A3 activity. Analysis of RNA-seq data from TCGA-BRCA showed no significant difference in ERBB2 expression between the A3+ and A3- groups, whereas FGFR4 expression was significantly higher in the A3+ group (Fig. S7e–f). This observation suggested a potential association between FGFR4 and A3 activity, prompting further evaluation of the effects of both genes on A3 expression and function.

BT-474 and MDA-MB-453 cell lines, which exhibit high expression of both ERBB2 and FGFR4 along with elevated A3 activity, were subjected to siRNA-mediated knockdown (Fig. 5d) [53]. In MDA-MB-453, both A3A and A3B expression were significantly reduced following FGFR4 knockdown, whereas no significant changes were observed after ERBB2 knockdown. A similar trend was observed in BT-474.

To assess functional consequences, an in vitro APOBEC3 deaminase activity assay was performed using the knockdown cell lines (Fig. 5e, f). In MDA-MB-453, mean DNA cleavage was 40.14% in controls, 35.08% after siERBB2, and 32.10% after siFGFR4. In BT-474, mean DNA cleavage was 62.51% in controls, 60.26% after siERBB2, and 57.03% after siFGFR4. This is consistent with the earlier observation that FGFR4 knockdown reduces A3 expression.

These results were corroborated by independent datasets: FGFR4 overexpression increased A3A and A3B expression, whereas pharmacological inhibition of FGFR4 suppressed both genes, with similar patterns observed in patient-derived xenografts (Fig. S10) [63]. Together, these findings suggest that FGFR4 may be a potential upstream regulator of A3 expression and activity.

## Discussion

In this study, we performed mutational signature analysis using the mutation profiles of human breast cancer (HBC) and canine mammary tumours (CMT), focusing on the distinct mutational landscapes revealed through cross-species comparison. We evaluated the extent to which CMT exhibits genomic landscapes similar to those of HBC in the context of cancer-associated mutations. Furthermore, to identify the factors contributing to mutational accumulation in HBC, we conducted an integrative genomic and transcriptomic analysis. Through this analysis, we aimed to identify the major drivers of mutational accumulation in HBC and to advance our understanding of their underlying mechanisms.

Our mutational signature analysis in HBC revealed a prominent presence of SBS2 and SBS13 signatures, which together accounted for nearly one-third of mutations in both whole-exome sequencing (WES) and whole-genome sequencing (WGS) data, as consistent with previous studies [21–25]. These signatures are attributed to APOBEC3 (A3)-mediated cytidine deamination, a process increasingly recognised as a potent driver of mutagenesis in breast cancer. Despite this prominent role of A3 in HBC, our mutational signature analysis revealed no evidence of SBS2 and SBS13 signatures in CMT.

Naturally occurring CMTs are recognised as potential translational models for HBC because they share several clinical and molecular features, including high prevalence, histopathological characteristics, comparable immunological landscapes, and specific mutational profiles [6–8]. Previous studies have specifically highlighted that the CMT model is characterised by a high frequency of the PIK3CA H1047R mutation, an oncogenic hotspot also prevalent in HBC [9]. However, our findings suggest that CMTs have limitations in recapitulating the complex genomic landscape of HBC, particularly regarding cancer-associated mutational processes. Previous studies have reported that CMTs often lack the downstream pathway activation seen in HBC and exhibit a substantially lower overall mutational burden [6, 64]. Furthermore, the absence of A3 activity in our analysis points toward mutational mechanisms in CMTs that are fundamentally distinct from those underlying the human disease, underscoring their interpretative limitations as a translational model for mutational processes.

Despite the mechanistic insights provided by comparative analysis, several technical and biological limitations must be acknowledged to ensure a balanced interpretation of these findings. First, the possibility of incomplete canine genome annotation and the interpretive constraints inherent in WES-based analysis may limit the comprehensive identification of mutational signatures. While the discovery of the ID83B signature points toward a canine-specific mutational process, its prevalence and biological significance require further validation against expanded canine-specific genomic libraries. Furthermore, although A3-associated signatures were absent in our cohort, the possibility remains that these processes occur at low frequencies in datasets not included in this study. More fundamentally, inherent biological disparities between species, such as differences in lifespan and hormonal exposure, could independently influence the analytical results. Specifically, as reported in previous studies, dogs have a significantly shorter lifespan than humans and exhibit a faster annual somatic mutation accumulation rate, suggesting that the underlying evolutionary mechanisms may differ inherently [65]. In addition, the timing of neutering and subsequent variations in hormone exposure in companion dogs represent critical factors that can exert independent effects on genomic instability. However, due to the retrospective nature of our large-scale cohort and the imbalanced distribution of clinical data, there were practical constraints in statistically adjusting for all potential confounding variables, including age, breed, and precise hormonal status. These considerations underscore that further investigations using stringently controlled prospective cohorts are essential to better elucidate the complex mutational mechanisms of CMTs, and that comparative genomic data must be interpreted with caution in light of these fundamental biological disparities. Consequently, rather than serving as universal models for all aspects of human breast cancer, CMTs should be more appropriately applied to studies with well-defined objectives. In particular, they may hold translational value in research targeting specific oncogenic pathways or therapeutic responses grounded in molecular similarities between the two species.

In our analysis, we primarily categorised the TCGA-HBC cohort into two groups based on the presence or absence of SBS2 and SBS13 signatures to elucidate the fundamental impact of A3 activation on the tumour genome. This binary classification allows for an intuitive understanding of key biological shifts by focusing on whether an A3-mediated event has occurred. However, such categorisation carries the inherent risk of failing to account for quantitative variability; by treating samples with low A3 activity (e.g., 5%) and high activity (e.g., 80%) as equivalent, this approach may fail to capture the nuances of progressive biological transitions from a statistical perspective. To complement these limitations of dichotomous analysis, we further performed an additional evaluation by defining A3 activity as a continuous variable. After controlling for extreme outliers using a Robust Linear Regression (RLM), we identified a significant correlation where each unit increase in A3-derived mutations was associated with a linear increase of approximately 0.14 in the total mutational burden (*P* = 0.009). Consistent with this, Spearman’s rank correlation (rho = 0.152, *P* < 0.0001) and global dose-response assessments across the A3 activity gradient further demonstrated that higher levels of A3 activity accelerate mutational accumulation (Fig. S11). Collectively, these multifaceted quantitative findings suggest that A3 does not merely play a role in inducing genetic mutations; rather, it has the potential to act as a pivotal driver that intensifies genetic complexity and heterogeneity in proportion to its activation strength.

A3 activity has been observed in a substantial proportion of breast cancer patients. To date, multiple upstream regulators of A3 expression have been identified at the molecular level, including interferons (IFNs), PKC/NFκB signalling, and DNA damage [21]. However, neither a definitive primary inducer nor consistent patient characteristics associated with elevated A3 activity have been clearly established. In this study, transcriptomic comparison of HBC patients with and without A3 activity revealed a strong association between A3 activity and the PAM50-defined HER2-Enriched (HER2E) subtype. Additionally, the knockdown of FGFR4 expression was found to significantly reduce the expression and enzymatic activity of both APOBEC3A and APOBEC3B. FGFR4 is one of the most prominent features of this subtype, and in contrast to ERBB2, its expression is also observed to a limited extent in other subtypes such as LumA and LumB [63]. Considering that A3 activity has been detected across multiple breast cancer subtypes, this broader expression pattern of FGFR4 further supports its possible involvement in the regulatory landscape of A3.

Although no direct regulatory relationship or feedback mechanism between A3 enzymes and FGFR4 has been reported to date, a mechanistic intersection between these factors remains biologically plausible. Recent large-scale genomic studies have established A3-mediated mutagenesis as a major driver of tumour evolution and therapy resistance in breast cancer, particularly in metastatic hormone receptor-positive disease [23, 24]. In parallel, FGFR4 has been implicated in subtype plasticity, luminal-to-HER2-Enriched transitions, and metastatic progression [63, 66]. While APOBEC3A and APOBEC3B are transcriptionally induced by pathways such as PKC/ncNFκB and JAK/STAT, FGFR4 activates signalling axes including PI3K/AKT and STAT3, which serve as central nodes in inflammatory and adaptive transcriptional programmes [21, 67]. Given that A3 expression and activity are frequently enhanced under conditions of inflammatory signalling and replication stress, sustained FGFR4 activation may create a permissive cellular environment that facilitates the upregulation or enzymatic activity of A3.

Furthermore, FGFR4-driven subtype plasticity entails extensive transcriptional reprogramming, a process associated with increased single-stranded DNA exposure during replication and transcription. Since A3 enzymes preferentially target such substrates, FGFR4-mediated plasticity may indirectly amplify A3 mutagenesis even in the absence of direct transcriptional control. Conversely, A3-driven genomic diversification under therapeutic pressure may facilitate the selection of clones that are increasingly dependent on adaptive signalling pathways such as FGFR4. Together, these findings suggest that FGFR4-driven plasticity and A3-mediated mutagenesis may cooperatively promote tumour evolution and therapy resistance. While our study utilised gene knockdown experiments, it remains limited in establishing a definitive causality, such as whether FGFR4 acts as a functional modulator or represents a highly correlative marker of the HER2E subtype. Additionally, direct mechanistic evidence, including in vivo validations, is currently lacking to fully support a more extensive interpretation of these findings. Therefore, further mechanistic studies using advanced experimental models will be necessary to clarify the precise nature of the potential regulatory link between FGFR4 and A3.

In conclusion, our study demonstrates that A3 mutagenesis is a defining feature of human breast cancer, characterised by markedly elevated activity in the HER2E subtype, and shows a potential regulatory association with FGFR4 signalling. These findings highlight a previously unrecognised association between FGFR4 and A3 that may contribute to the aggressive nature of HER2E tumours and suggest that this axis merits potential integration into the molecular biomarker framework for the classification of the HER2E subtype. At the same time, the absence of A3 activity in canine mammary tumours underscores important interspecies differences and highlights the need in comparative oncology to select models that are appropriate for clearly defined research objectives.

## Methods

### Human breast cancer mutation matrix acquisition

Mutation count matrices used for mutational signature analysis in human breast cancer were initially obtained from Synapse and the International Cancer Genome Consortium (ICGC) [4]. Following the closure of the ICGC Data Portal in June 2024, data were accessed via Amazon Web Services (AWS) using the S3 interface. SBS96 and ID83 matrices from whole-genome sequencing (PCAWG) and whole-exome sequencing (TCGA) were downloaded. Datasets were filtered to include only breast cancer samples. After filtering, 930 TCGA samples (Breast Invasive Carcinoma) and 214 PCAWG samples (Breast Adenocarcinoma, *n* = 198; Breast Lobular Carcinoma, *n* = 13; Ductal Carcinoma In Situ, *n* = 3) were included.

### WES analysis of canine mammary tumours

A whole-exome sequencing dataset of canine mammary tumours and matching adjacent normal tissues was used in this study. The dataset comprised a total of 205 pairs: 185 pairs from Yonsei University and 20 pairs from CDRC [9, 10]. The original sample collection and data generation were conducted with informed owner consent and under the ethical approval of the Institutional Animal Care and Use Committees (IACUC) of Konkuk University (KU16106, KU17162) and Seoul National University (IACUC# SNU-170602-1). All procedures were performed in accordance with the relevant institutional guidelines and ethical regulations for animal research. Clinical features of each sample, including ID, breed, age, sex, spay, as well as cancer type category, are listed in Table S1.

Raw sequencing data were reanalysed to improve detection accuracy and incorporate the latest gene annotation tools. Sequencing data (.fastq.gz) were downloaded from the NCBI Sequence Read Archive (SRA) and European Nucleotide Archive (ENA). Specifically, the following SRR accession numbers were included (SRR9911342-SRR9911381; SRR7780731-SRR7781112; http://www.ncbi.nlm.nih.gov/sra/). Note that the latter range includes some samples that are not part of the final 205 paired CMT dataset.

The Sequence reads were aligned to the CanFam3.1 reference genome (https://ftp.ensembl.org/pub/release-104/) using BWA-MEM (v0.7.18), and output files were generated in SAM file format [11]. To ensure compatibility with downstream analysis tools, SAM files were converted into BAM format using HTSlib (v1.20) [12]. PCR duplicates were marked and removed using Picard’s MarkDuplicates (v3.2.0) to improve data quality [13]. Base quality score recalibration was then performed using the Genome Analysis Toolkit (GATK; v4.5.0.0) to ensure that quality scores more accurately reflect actual error rates [14].

Tumour-specific somatic mutations were called using Strelka2 (v2.9.10), with outputs generated in VCF format [15]. To annotate the biological impact of each mutation, VCF files were converted to MAF format using VCF2MAF (v1.6.21), which incorporates transcript-aware annotations, predicted functional impact, and gene-level context from Ensembl VEP to facilitate downstream analysis [16].

All parameters were set by default. Mapping quality and coverage depth of the sequencing data are listed in Table S2. Variant information within coding regions is listed in Table S3.

### Analysis of mutational signatures with SigProfiler tools

Somatic mutation count matrices for single base substitution (SBS) and small insertion and deletion (ID) were generated from VCF files using SigProfilerMatrixGenerator (v1.2.28) [17]. De novo mutational signatures were extracted using SigProfilerExtractor (v1.1.24), which implements mathematical models based on non-negative matrix factorisation (NMF) [18]. This algorithm is designed to derive de novo signatures that provide the most accurate representation of the underlying mutational complexity within the patient dataset. The NMF process was initialised using the default “random” initialisation method, with convergence defined by a tolerance threshold of 1 × 10^-15^ and a maximum of 1,000,000 internal iterations to ensure high-precision decomposition. To identify the optimal number of de novo signatures, models were evaluated across a range of 1 to 10 candidate solutions, with 500 NMF replicates performed per configuration to ensure robustness. This extensive iteration process serves to isolate genuine mutational signals from stochastic noise, thereby ensuring the extraction of stable and reproducible patterns. The optimal number of signatures was determined by evaluating the trade-off between the stability of the solution and the reconstruction error, specifically by minimising the Kullback-Leibler divergence and selecting an average Silhouette width (stability threshold) of ≥ 0.80. As detailed in Table S4, the final solution was selected based on achieving the highest stability alongside a minimised Matrix Frobenius reconstruction error. The extracted signatures were assigned to the COSMIC database (COSMIC_v3_GRCh37; https://cancer.sanger.ac.uk/cosmic/signatures) using SigProfilerAssignment (v0.1.8) to assess their similarity to established human cancer mutational patterns [19]. This comparative framework leverages the vast repository of tens of thousands of human cancer profiles to provide an objective basis for inferring and characterising the underlying mutational processes identified in our tumour samples. Solution statistics and evaluation for SBS96 and ID83 matrices, are listed in Table S4.

### TCGA-BRCA WES and RNA-seq data acquisition

Whole-exome sequencing (WES) and RNA-sequencing (RNA-seq) data for TCGA-BRCA were obtained from the Genomic Data Commons (GDC) Portal (https://portal.gdc.cancer.gov/). Patient IDs for 930 individuals were extracted and matched to corresponding sample IDs to retrieve the relevant WES mutation profiles and RNA-seq expression data. To capture the fundamental impact of APOBEC3 (A3) activity on tumour characteristics, patients were conservatively stratified based on the binary presence of A3-associated mutational signatures (SBS2 or SBS13), rather than utilising specific exposure cutoffs. The HBC(A3 + ) group was defined by the detection of at least one mutation attributed to either signature, while the HBC(A3-) group included those with neither. For mutation data, patients without available mutation profiles were excluded, resulting in 374 HBC(A3 + ) samples and 496 HBC(A3-) samples. Variants lacking proper annotation were also filtered out and excluded from downstream analyses. For RNA-seq data, patients without corresponding expression datasets were excluded, resulting in 401 HBC(A3 + ) and 526 HBC(A3-) samples. For the quantitative comparison of gene expression across patients, log₂(TPM + 1)-transformed values were utilised. The normality of the data distribution was evaluated using the D’Agostino-Pearson omnibus normality test. Given that the data did not meet the assumptions of a normal distribution (*p* ≤ 0.05), non-parametric statistical methods were employed. Specifically, the Kruskal–Wallis test was used to assess differences in gene expression across groups. To account for the increased risk of Type I errors associated with multiple comparisons, post hoc pairwise evaluations were performed using Dunn’s multiple comparison test, with *P*-values adjusted accordingly to maintain statistical stringency. Statistical tests were two-sided, and a *P*-value < 0.05 was considered to indicate statistical significance.

### Identification and classification of hotspot mutations

Hotspot mutations among the called variants in CMT and HBC were identified based on previously described criteria (https://www.cancerhotspots.org/#/home) [34]. Recurrently mutated sites within A3-associated contexts and statistically significant mutation hotspots (Q value < 0.05) were selected. The identified hotspots were subsequently classified according to A3 mutational status and are listed in Table S6.

### Comparative sequence and structural analysis of APOBEC3 proteins

Sequence similarity between human and canine A3 was assessed using the Basic Local Alignment Search Tool (BLAST) provided by NCBI (https://blast.ncbi.nlm.nih.gov/Blast.cgi). To evaluate interspecies structural similarity of A3 proteins, the corresponding PDB-format structures were retrieved from the AlphaFold Protein Structure Database (https://alphafold.ebi.ac.uk/). These AlphaFold2-predicted models were used directly for comparative analysis, as they had already undergone stereochemical refinement via the default AMBER relaxation pipeline within the AlphaFold2 framework. Pairwise structural comparisons were performed using the TM-align algorithm (https://zhanggroup.org/TM-align/), which calculates the root mean square deviation (RMSD) and TM-score between two protein structures. Similarity metrics were normalised to the length of the corresponding canine ortholog. As controls, protein structures of the four most frequently mutated genes in TCGA-BRCA (TP53, CDH1, PIK3CA, and PTEN) were included, based on mutation frequency data from the GDC data portal.

Sequence alignment of A3 proteins from human and canine was performed using Clustal Omega (https://www.ebi.ac.uk/jdispatcher/msa/clustalo), and the resulting alignment was used to visualise secondary structure features using ESPript (https://espript.ibcp.fr/ESPript/ESPript/).

Protein structure visualisation was performed using UCSF ChimeraX (https://www.cgl.ucsf.edu/chimerax/). To evaluate the confidence of the predicted 3D structures, protein sequences were retrieved from UniProt (https://www.uniprot.org/). Each sequence was processed through the AlphaFold server (https://alphafoldserver.com/) to generate pLDDT model files (models 0-4). For each model, the average pLDDT score of the backbone atoms (C, N, O, and Cα) was calculated for all amino acid residues, and the model with the highest mean pLDDT score was selected for visualisation. This multi-model approach ensured that the structural predictions were consistent across independent simulations. All proteins were structurally aligned based on the positions of loop 1, 3, and 7 regions. The reliability of this alignment was further validated by confirming that most functional loop regions maintained high confidence scores (e.g., pLDDT > 70), ensuring that the comparative analysis prioritised high-confidence structural coordinates. In addition, Predicted Aligned Error (PAE) data were downloaded from the AlphaFold Protein Structure Database (https://alphafold.ebi.ac.uk/) to cross-validate the structural confidence levels and the stability of relative domain orientations of each protein.

### Cell culture

Cryopreserved stocks of canine mammary gland adenocarcinoma and human breast cancer cell lines, previously obtained from collaborators, were thawed and cultured for subsequent experiments. All cell lines were previously established and provided by collaborators for research purposes, and their use was conducted in accordance with the institutional ethical guidelines for established cell lines. CHMp, CHMm, CIPp, CIPm, and CTBp cells were maintained in RPMI-1640 medium (SH30027.01, Hyclone) supplemented with 0.5% gentamicin (G1272, Sigma). Non-tumorigenic MCF-10A cells were cultured in MEGM BulletKit (CC-3151 and CC-4136, Lonza). MCF-7, MDA-MB-453, MDA-MB-231, and HS578T cells were grown in high-glucose DMEM (SH30243.01, Hyclone), while BT-474, T-47D, and SK-BR-3 cells were maintained in RPMI-1640 medium. All media were supplemented with 10% fetal bovine serum (1600044, Gibco, MA, USA) and 1% antibiotic-antimycotic solution (15240-062, Gibco, MA, USA). Cells were incubated at 37 °C in a humidified atmosphere containing 5% CO₂, and all cell lines were confirmed to be free of mycoplasma contamination.

### RNA isolation and quantitative PCR

RNA isolation, quantification, and quantitative RT-PCR were performed as described previously [53]. Briefly, reverse transcription was performed using the CellScript All-in-One 5X 1st cDNA strand synthesis Master Mix (CDS-400, Cellsafe). cDNA synthesis reactions were performed using 1 µl of RNA (50 ng/µl), 2 µl of CellScript, and DEPC-treated water to a total volume of 20 µl. Relative mRNA expression levels were quantified using the comparative ΔΔCt method, with GAPDH employed as the internal control for normalisation. Differences across groups were assessed using the two-sided ordinary one-way ANOVA, with Tukey’s multiple comparison test applied for post hoc pairwise evaluations. Data are presented as means ± S.D. from a representative experiment conducted in technical triplicates. The reproducibility of the findings was confirmed through at least three independent biological replicates with similar results. All primer sequences are listed in Table S7.

To validate the specificity and size of the qPCR amplicons, electrophoresis was performed on a 2% agarose gel (A0224-050, GenDEPOT) prepared in TAE buffer containing SYBR Safe DNA Gel Stain (s33102, invitrogen). DNA samples were mixed with TriTrack DNA Loading Dye(6X) (R1161, Thermo) prior to loading, and a 100 bp DNA ladder was used as a size reference.

### In vitro DNA deaminase activity assay

APOBEC3 deamination activity assays were performed based on previously described methods [23]. 2 million cells were pelleted and lysed in buffer (25 mM HEPES, 200 mM NaCl, 10% glycerol, 1 mM MgCl2, 0.5% Triton X-100, 1× protease inhibitor cocktail, 1 mM EDTA), sheared through a 29 G Insulin Syringe (BD Ultra-Fine), then cleared by centrifugation at 13,000 g for 10 min at 4 °C. Deaminase reactions (16.5 µl cell extracts with 2 µl UDG buffer (NEB), 0.5 µl RNase A (20 mg ml^-^1), 1 µl of 1 µM oligonucleotide probe, and 0.3 µl UDG (NEB) were incubated at 37 °C for 2 h followed by addition of 2 µl 1 M NaOH and 15 min at 95 °C to cleave abasic sites. Reactions were then neutralised with 2 µl 1 M HCl, terminated by adding 20 µl formamide buffer (90% formamide + EDTA) and separated on a 15% PAGE gel in 1× TBE buffer at 80 min at 110 V to monitor DNA cleavage. Gels were imaged by GelDoc Go Gel Imaging System and quantified using imageJ [56]. Statistical significance was determined using the two-sided ordinary two-way ANOVA followed by Tukey’s multiple comparison test to account for interaction effects between substrates and groups. Data are presented as means ± S.D. from a representative experiment conducted in technical triplicates. The reproducibility of the findings was confirmed through at least three independent biological replicates with similar results. All oligonucleotide probe sequences are listed in Table S7.

### Differential gene expression analysis

To investigate transcriptomic differences associated with APOBEC activity, TCGA-BRCA patients were stratified into two groups: HBC(A3 + ) (*n* = 401) and HBC(A3-) (*n* = 526). RNA-seq data for each TCGA-BRCA patient ID were retrieved, and TPM-normalised expression values were used for analysis. Because TPM values represent relative proportions normalised across samples, they can distort absolute expression differences and fail to adequately capture biological variability. To stabilise variance and approximate normality of the data distribution, TPM values were transformed using log₂(TPM + 1). Based on these transformed values, a linear modelling approach was implemented using the limma package in R, which applies empirical Bayes moderation to borrow information across genes and improve variance estimation. Mean expression differences between the two groups were mathematically estimated using two-sided tests, and statistical reliability was further enhanced by adjusting gene-wise variances with a common variance estimated across all genes. To control the false discovery rate (FDR) in multiple hypothesis testing, P-values were adjusted using the Benjamini–Hochberg (BH) method. Genes with an adjusted *P* < 0.05 were considered significantly differentially expressed (DEGs). For these identified DEGs, the log_2_ fold change (log_2_FC) was utilised as the primary measure of effect size to represent the magnitude of transcriptomic shifts. Rather than applying an arbitrary log_2_FC threshold, we prioritised all statistically significant genes for downstream functional interpretation and visualisation to ensure a comprehensive capture of A3-related biological variability.

Significant DEGs were visualised in a heatmap using the pheatmap package in R. To maintain the biological context of the clinical groupings, samples (columns) were manually ordered according to their “PAM50 and Claudin-low (CLOW) molecular subtype” and “A3 activity” classification, with column clustering disabled (cluster_cols = FALSE). To highlight relative expression patterns across the stratified groups, hierarchical clustering was performed on the genes (rows) using Euclidean distance and the Ward.D2 linkage method. For visualisation, expression values were scaled via row-wise Z-score normalisation to emphasise gene-specific expression trends regardless of absolute transcript abundance.

### Cell line expression data processing

PAM50 gene set-based expression data (Batch-corrected Expression Public 24Q4) were obtained from the DepMap portal (https://depmap.org/portal). Breast cancer cell lines were selected from this dataset for analysis. Gene-wise expression patterns were compared using Euclidean distance-based clustering, and Z-score normalisation was applied to expression values across genes. Cell subtype annotations provided by DepMap were visualised and compared with the clustering results.

To evaluate transcriptional similarity, correlation coefficients were calculated between each cell line’s gene expression profile and the reference PAM50 subtype signatures (Table S9) [68]. Each cell line was assigned to the PAM50 subtype with the highest correlation coefficient, and the probability distribution of the subtype was also computed.

### siRNA transfection

siRNA transfection were processed as described previously methods [53]. Negative control siRNA, ERBB2 siRNA, and FGFR4 siRNA were obtained from Bioneer (Daejeon, Republic of Korea). Pre-designed ERBB2 and FGFR4 siRNAs (siRNA ID: 2064-2 and 2264-2, respectively) were used for knockdown experiments. Transfections were carried out using Lipofectamine RNAiMAX Transfection Reagent (Cat. No. 13778150, Invitrogen, MA, USA) following the manufacturer’s instructions (Protocol No. MAN0007825). For transfection, MDA-MB-453 and BT-474 cells were seeded in 6-well plates at a density of 0.5 × 10⁶ and 1.0 × 10⁶ cells per well, respectively. After 16 hours of incubation,diluted siRNA complexes were added to the cells. Specifically, 3 µL (30 pmol) of ERBB2 siRNA and 4 µL (40 pmol) of FGFR4 siRNA were used per well. Cells were harvested 24 hours post-transfection for RNA extraction and gene expression analysis. In parallel, APOBEC3 activity assays were conducted using cells collected at the same time point. To ensure reproducibility, all experiments were performed as at least three independent biological replicates. Representative data from a single experiment, analysed in technical triplicates, are presented as means ± SD for two-group comparisons and means ± SEM for three-group comparisons. Statistical significance between two groups was assessed using a two-sided paired *t*-test. For multiple comparisons involving three groups, a two-sided ordinary one-way ANOVA followed by Tukey’s multiple comparison test was employed to account for multiple testing.

## Supplementary information

Supplementary Information

Supplementary Figure S1-S11

Supplementary Table S1-S9

Movie 1. 3D structural alignment of canine APOBEC3Z1 and human APOBEC3A

Movie 2. 3D structural alignment of canine APOBEC3Z1 and human APOBEC3B

Movie 3. 3D structural alignment of canine APOBEC3Z1 and human APOBEC3G

Movie 4. 3D structural alignment of canine APOBEC3Z3 and human APOBEC3H

Reproducibility Checklist

## Data Availability

All data sources or processed datasets supporting the findings of this study are included in the paper and its Supplementary Information. Processed data are available from the corresponding author upon reasonable request.

## Code availability

Code is available from the corresponding author upon reasonable request.
