# Supplementary material for: FGFR4-associated APOBEC3 mutagenesis characterizes HER2-enriched subtype of human breast cancer
Source: Br J Cancer. 2026 Jul 27;135(9):1483–98. doi: 10.1038/s41416-026-03517-8 (PMC13675202; doi:10.1038/s41416-026-03517-8)
Supplement: Supplementary file 1 [file 41416_2026_3517_MOESM2_ESM.pdf]

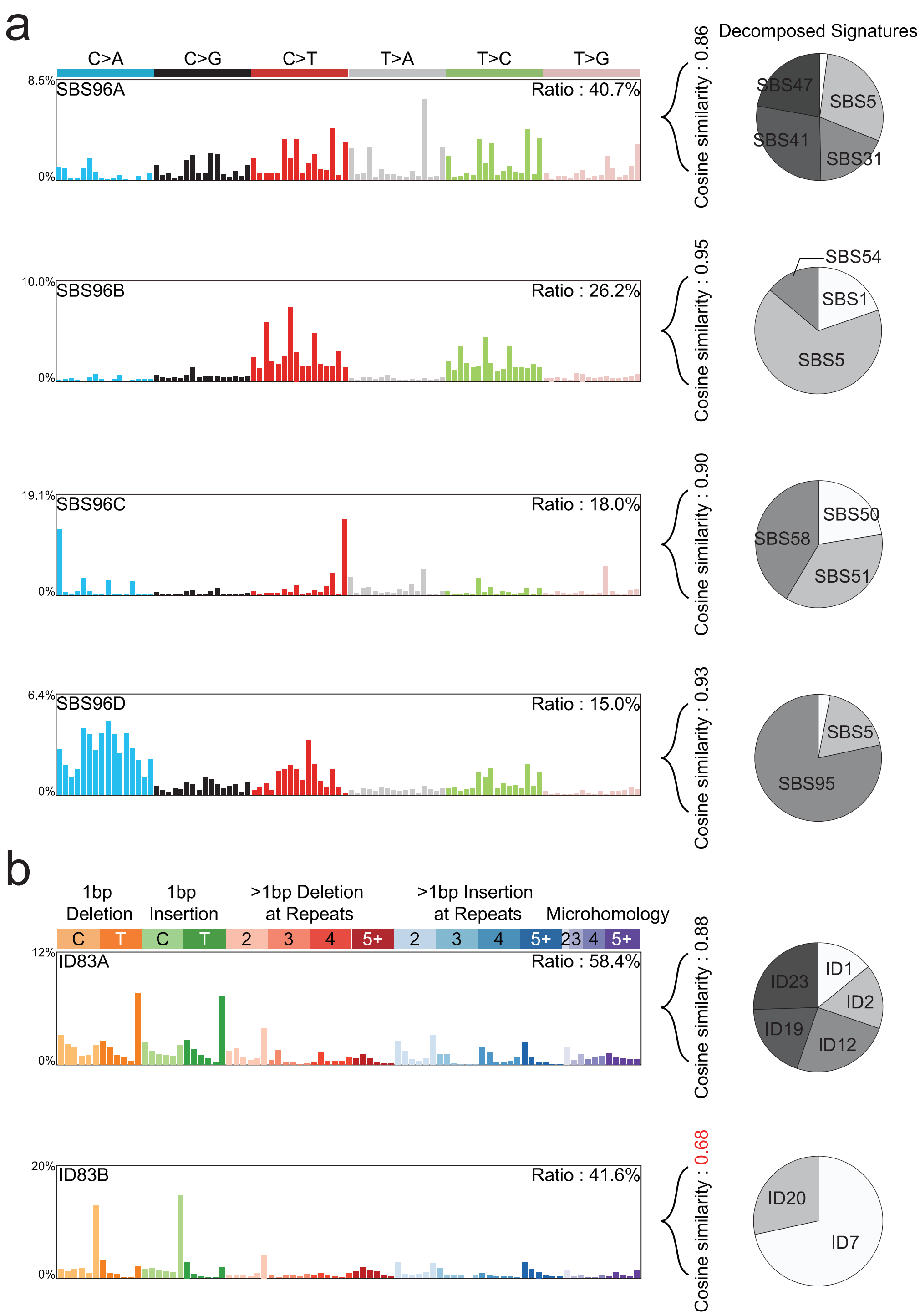
a
C>A
C>G
C>T
T>A
T>C
T>G
Decomposed Signatures
8.5%
0%
SBS96A
Ratio : 40.7%
Cosine similarity : 0.86
SBS47
SBS5
SBS41
SBS31
10.0%
0%
SBS96B
Ratio : 26.2%
Cosine similarity : 0.95
SBS54
SBS1
SBS5
19.1%
0%
SBS96C
Ratio : 18.0%
Cosine similarity : 0.90
SBS50
SBS58
SBS51
6.4%
0%
SBS96D
Ratio : 15.0%
Cosine similarity : 0.93
SBS5
SBS95
b
1bp Deletion
1bp Insertion
>1bp Deletion at Repeats
>1bp Insertion at Repeats
Microhomology
C
T
C
T
2
3
4
5+
2
3
4
5+
2
3
4
5+
12%
0%
ID83A
Ratio : 58.4%
Cosine similarity : 0.88
ID1
ID23
ID2
ID19
ID12
20%
0%
ID83B
Ratio : 41.6%
Cosine similarity : 0.68
ID20
ID7

a

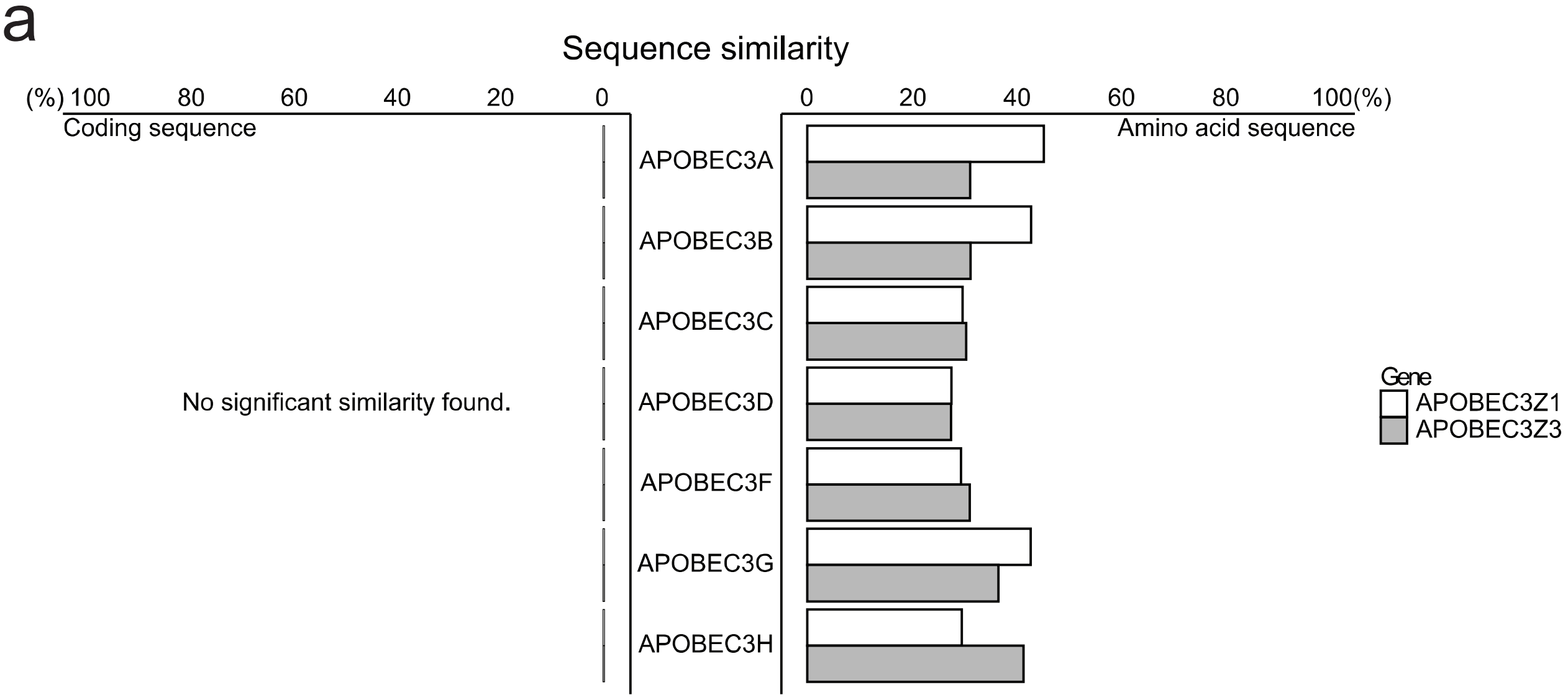
Sequence similarity
(%) 100
80
60
40
20
0
0
20
40
60
80
100(%)
Coding sequence
Amino acid sequence
APOBEC3A
APOBEC3B
APOBEC3C
APOBEC3D
APOBEC3F
APOBEC3G
APOBEC3H
No significant similarity found.
Gene
APOBEC3Z1
APOBEC3Z3

b

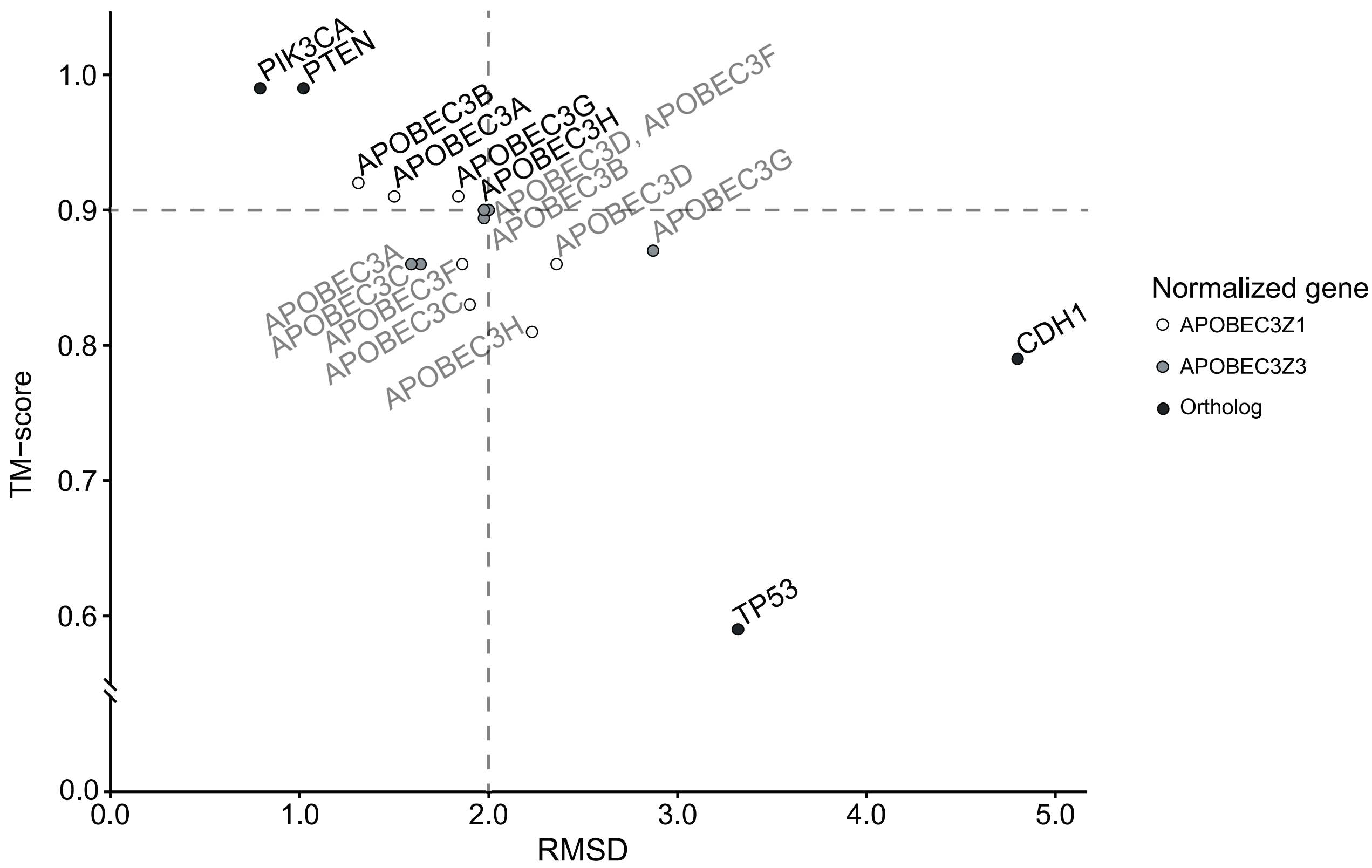
TM-score
RMSD
1.0
0.9
0.8
0.7
0.6
0.0
0.0
1.0
2.0
3.0
4.0
5.0
PIK3CA
PTEN
APOBEC3B
APOBEC3A
APOBEC3G
APOBEC3H
APOBEC3D, APOBEC3F
APOBEC3B
APOBEC3D
APOBEC3G
APOBEC3A
APOBEC3C
APOBEC3F
APOBEC3C
APOBEC3H
CDH1
TP53
Normalized gene
APOBEC3Z1
APOBEC3Z3
Ortholog

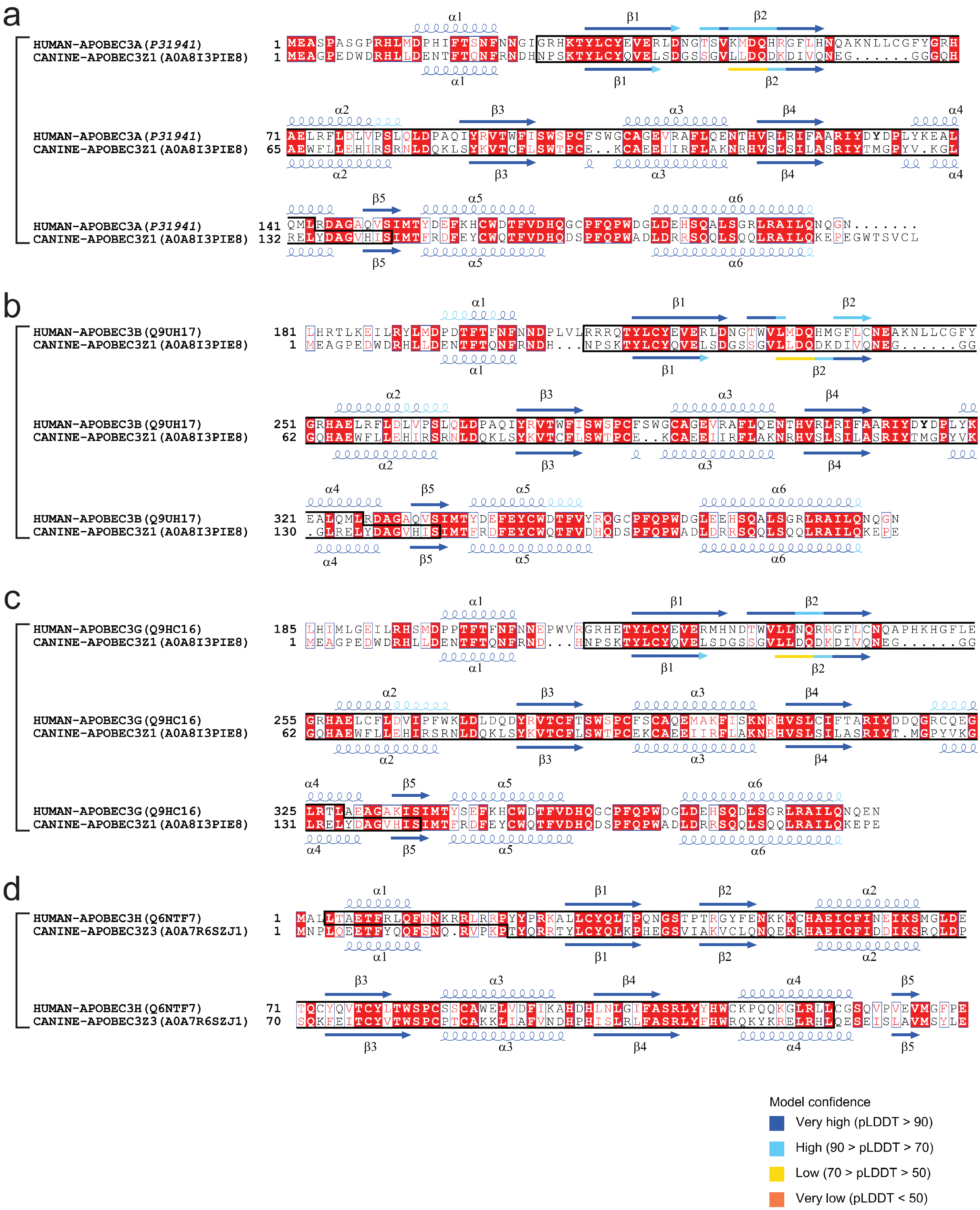
a
HUMAN-APOBEC3A (P31941)
CANINE-APOBEC3Z1 (A0A8I3PIE8)
b
HUMAN-APOBEC3B (Q9UH17)
CANINE-APOBEC3Z1 (A0A8I3PIE8)
c
HUMAN-APOBEC3G (Q9HC16)
CANINE-APOBEC3Z1 (A0A8I3PIE8)
d
HUMAN-APOBEC3H (Q6NTF7)
CANINE-APOBEC3Z3 (A0A7R6SZJ1)
Model confidence
Very high (pLDDT > 90)
High (90 > pLDDT > 70)
Low (70 > pLDDT > 50)
Very low (pLDDT < 50)

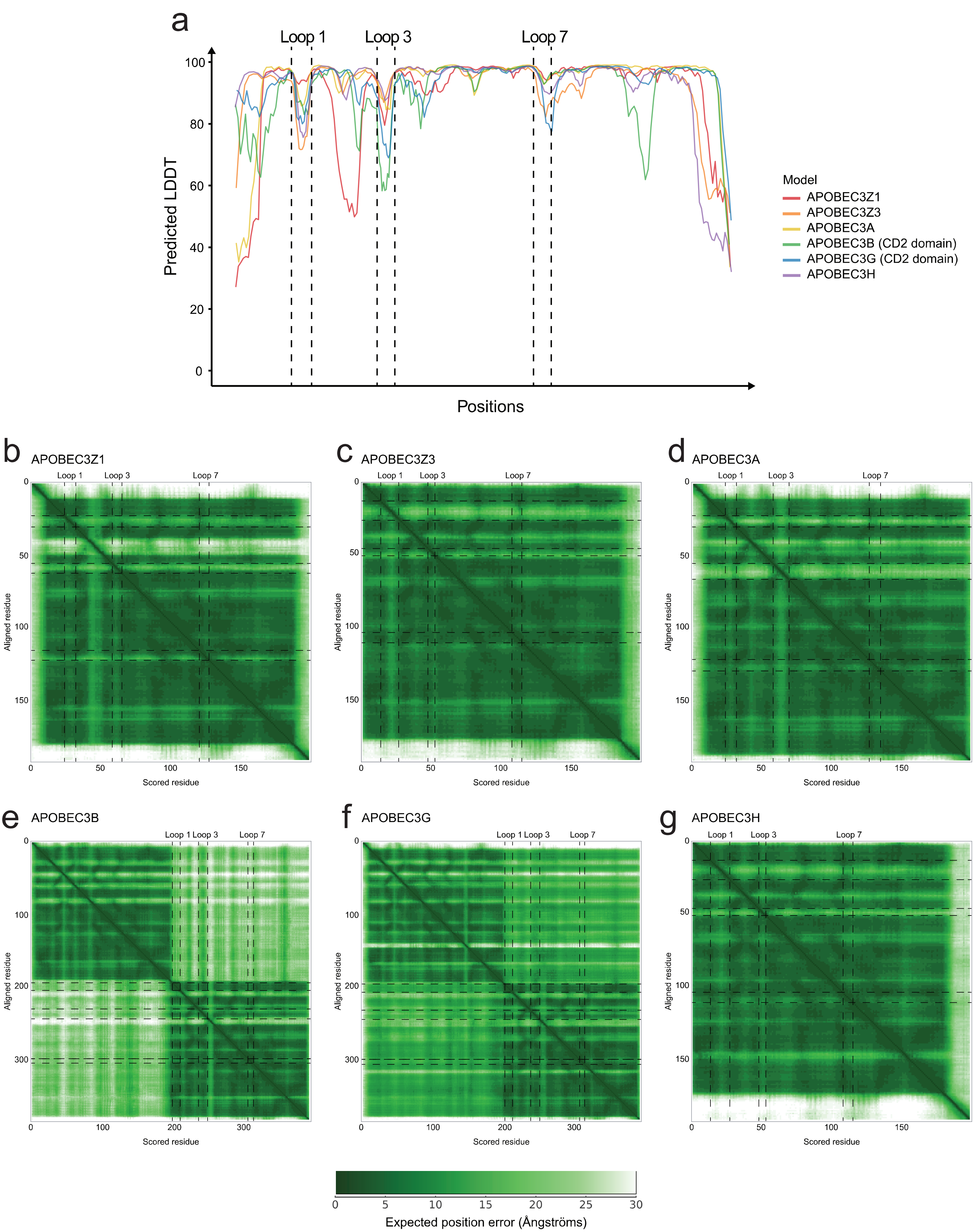

a
Loop 1
Loop 3
Loop 7
Predicted LDDT
Positions
Model
APOBEC3Z1
APOBEC3Z3
APOBEC3A
APOBEC3B (CD2 domain)
APOBEC3G (CD2 domain)
APOBEC3H
b APOBEC3Z1
c APOBEC3Z3
d APOBEC3A
e APOBEC3B
f APOBEC3G
g APOBEC3H
Aligned residue
Scored residue
Expected position error (Ångströms)

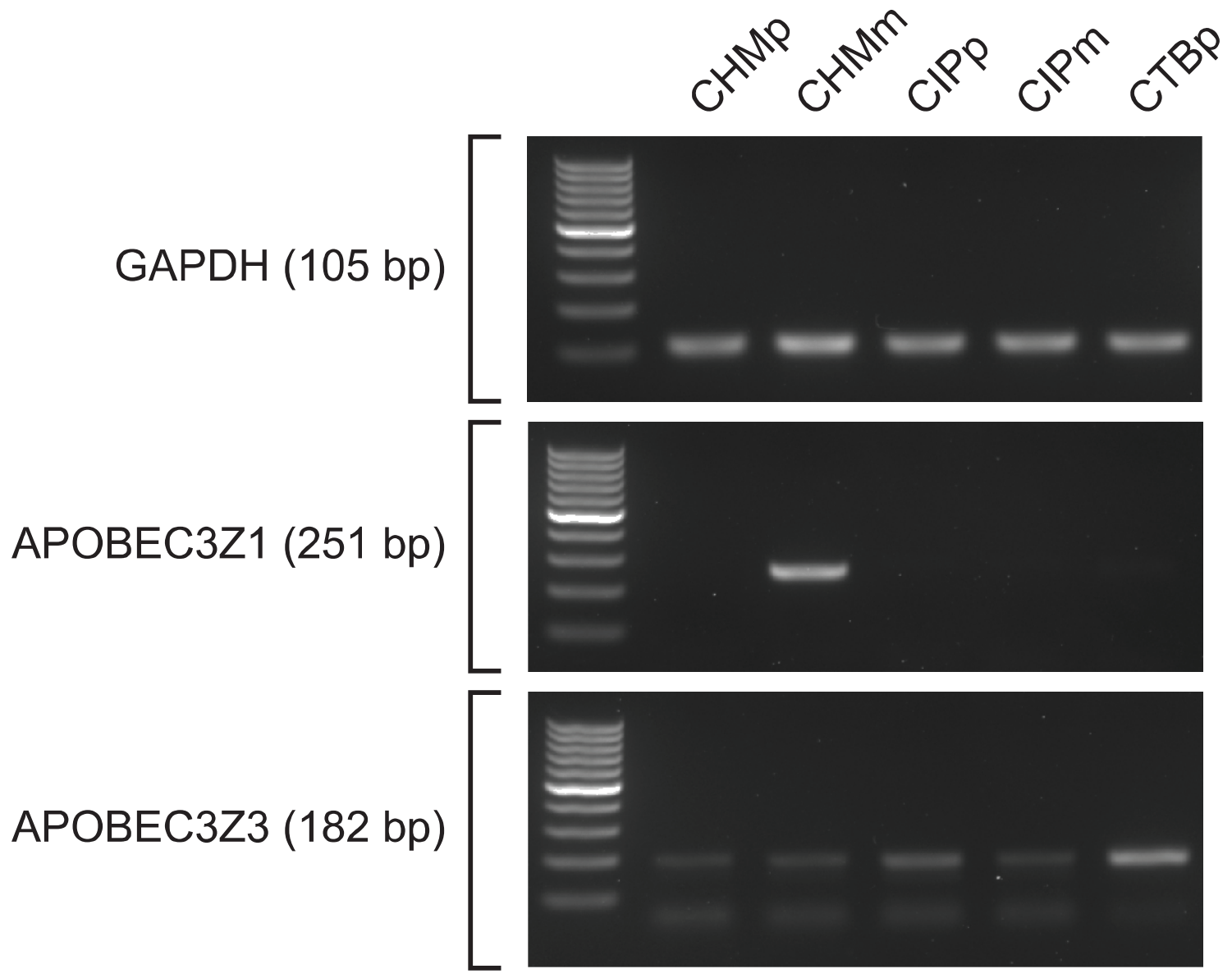
CHMp
CHMm
CIPp
CIPm
CTBp
GAPDH (105 bp)
APOBEC3Z1 (251 bp)
APOBEC3Z3 (182 bp)

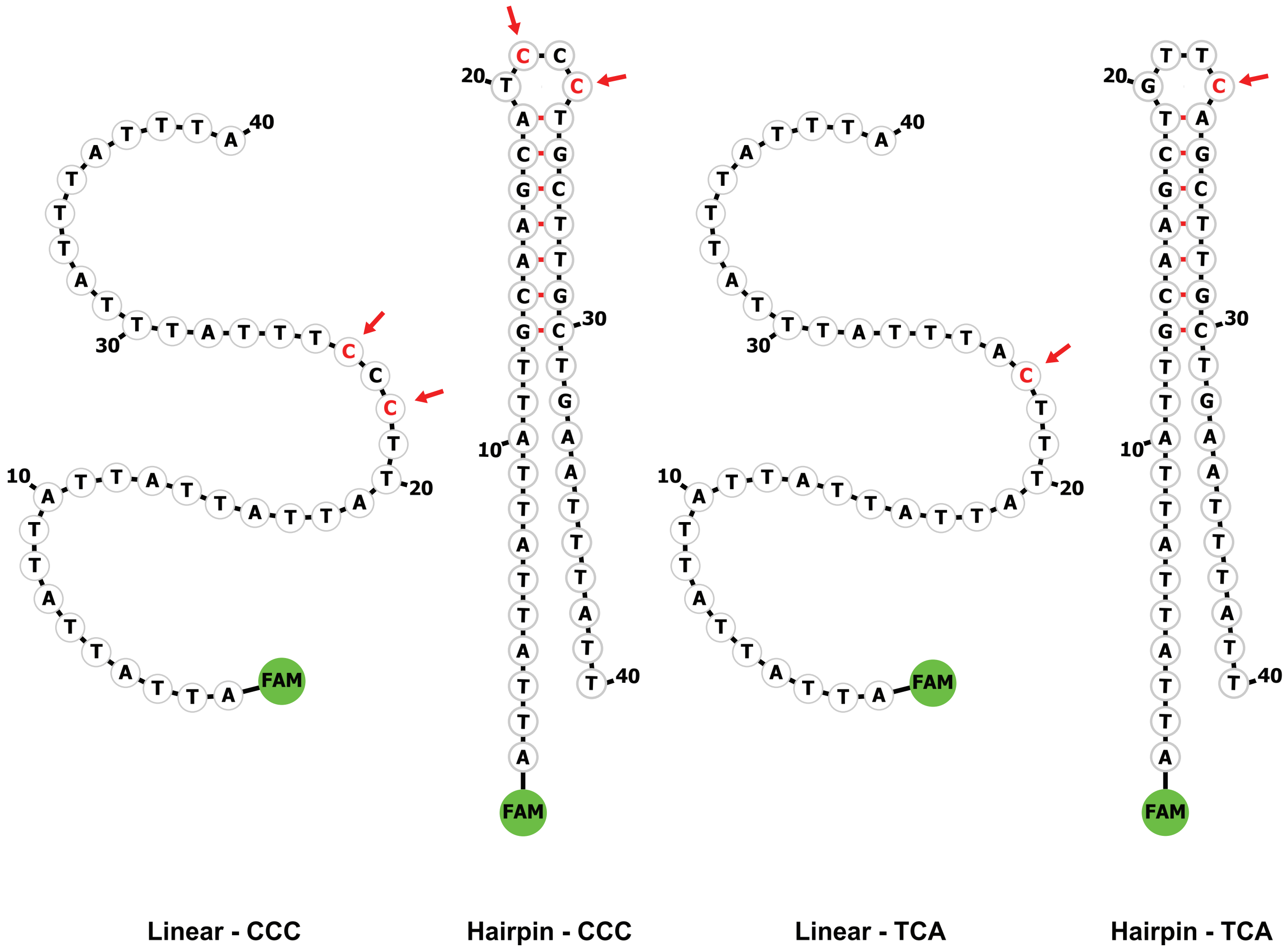

FAM
Linear - CCC
Hairpin - CCC
Linear - TCA
Hairpin - TCA

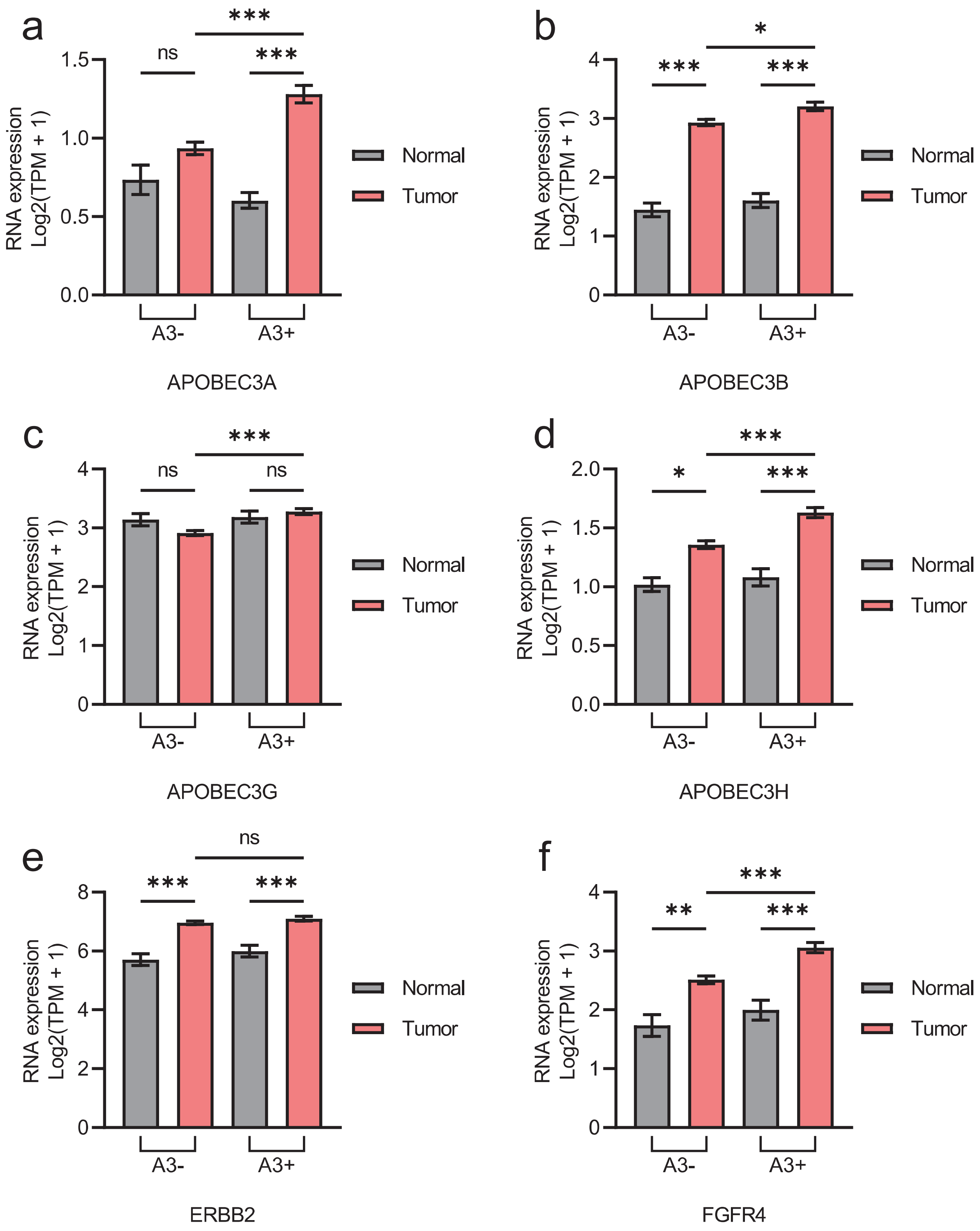

a
***
ns
***
1.5
1.0
0.5
0.0
RNA expression
Log2(TPM + 1)
A3-
A3+
APOBEC3A
Normal
Tumor
b
*
***
***
4
3
2
1
0
RNA expression
Log2(TPM + 1)
A3-
A3+
APOBEC3B
Normal
Tumor
c
***
ns
ns
4
3
2
1
0
RNA expression
Log2(TPM + 1)
A3-
A3+
APOBEC3G
Normal
Tumor
d
***
*
***
2.0
1.5
1.0
0.5
0.0
RNA expression
Log2(TPM + 1)
A3-
A3+
APOBEC3H
Normal
Tumor
e
ns
***
***
8
6
4
2
0
RNA expression
Log2(TPM + 1)
A3-
A3+
ERBB2
Normal
Tumor
f
***
**
***
4
3
2
1
0
RNA expression
Log2(TPM + 1)
A3-
A3+
FGFR4
Normal
Tumor

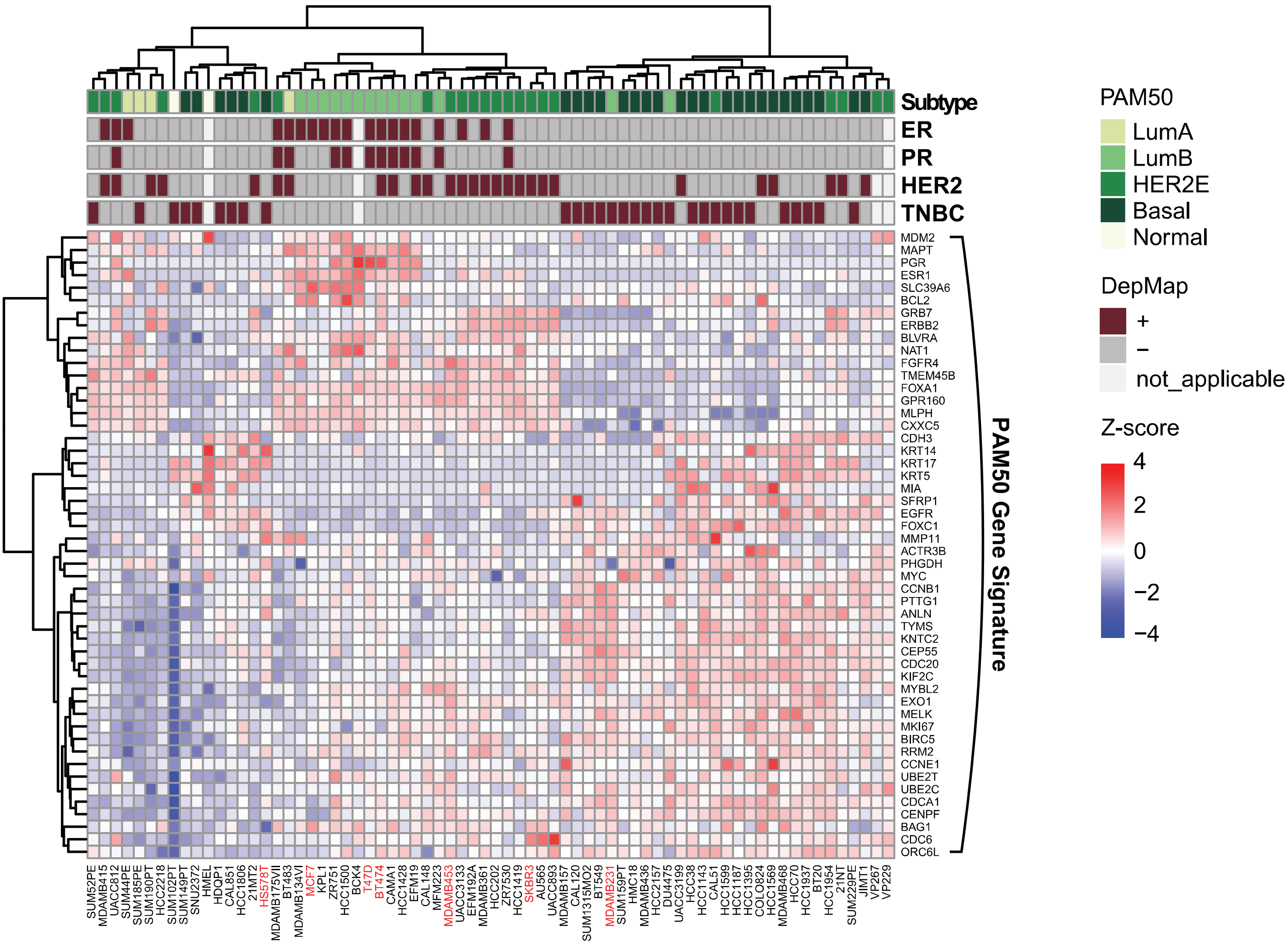

Subtype
ER
PR
HER2
TNBC
PAM50
LumA
LumB
HER2E
Basal
Normal
DepMap
+
−
not_applicable
Z-score
4
2
0
−2
−4
PAM50 Gene Signature
MDM2
MAPT
PGR
ESR1
SLC39A6
BCL2
GRB7
ERBB2
BLVRA
NAT1
FGFR4
TMEM45B
FOXA1
GPR160
MLPH
CXXC5
CDH3
KRT14
KRT17
KRT5
MIA
SFRP1
EGFR
FOXC1
MMP11
ACTR3B
PHGDH
MYC
CCNB1
PTTG1
ANLN
TYMS
KNTC2
CEP55
CDC20
KIF2C
MYBL2
EXO1
MELK
MKI67
BIRC5
RRM2
CCNE1
UBE2T
UBE2C
CDCA1
CENPF
BAG1
CDC6
ORC6L
SUM52PE
MDAMB415
UACC812
SUM44PE
SUM185PE
SUM190PT
HCC2218
SUM102PT
SUM149PT
SNU2372
HMEL
HDQP1
CAL851
HCC1806
21MT2
HS578T
MDAMB175VII
BT483
MDAMB134VI
MCF7
KPL1
ZR751
HCC1500
BCK4
T47D
BT474
CAMA1
HCC1428
EFM19
CAL148
MFM223
MDAMB453
UACC3133
EFM192A
MDAMB361
HCC202
ZR7530
HCC1419
SKBR3
AU565
UACC893
MDAMB157
CAL120
SUM1315MO2
BT549
MDAMB231
SUM159PT
HMC18
MDAMB436
HCC2157
DU4475
UACC3199
HCC38
HCC1143
CAL51
HCC1599
HCC1187
HCC1395
COLO824
HCC1569
MDAMB468
HCC70
HCC1937
BT20
HCC1954
21NT
SUM229PE
JIMT1
VP267
VP229

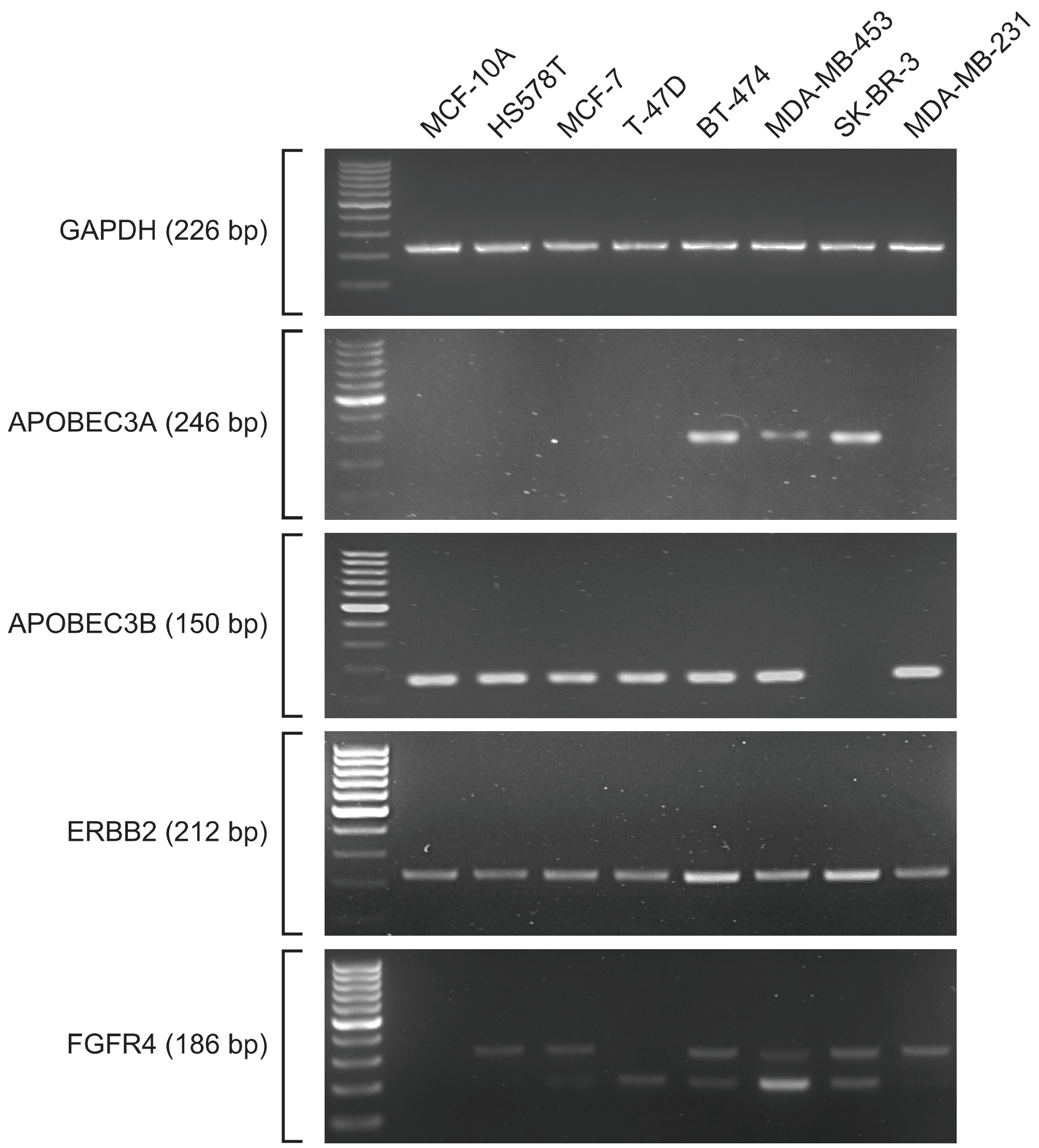
MCF-10A
HS578T
MCF-7
T-47D
BT-474
MDA-MB-453
SK-BR-3
MDA-MB-231
GAPDH (226 bp)
APOBEC3A (246 bp)
APOBEC3B (150 bp)
ERBB2 (212 bp)
FGFR4 (186 bp)

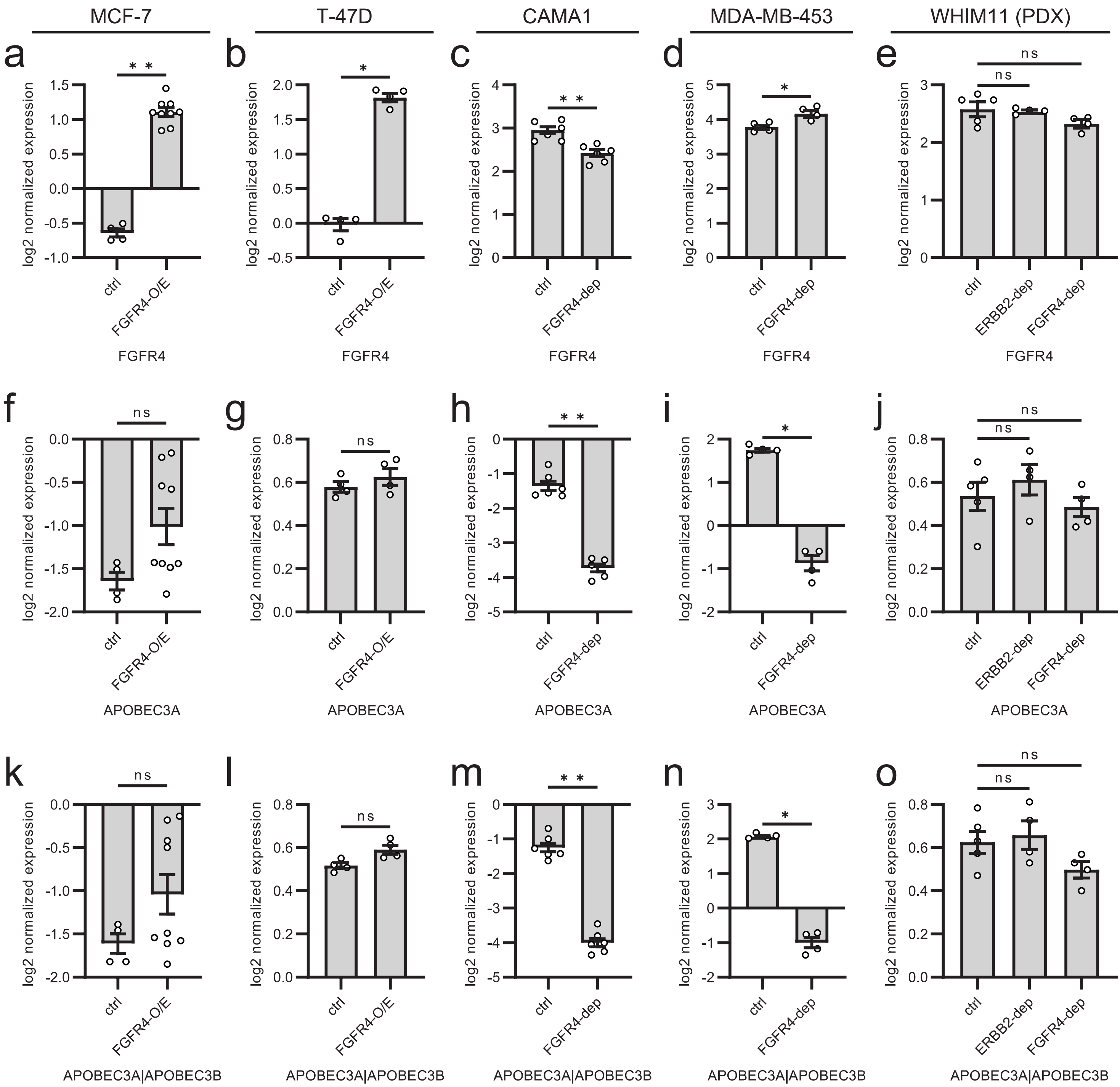
MCF-7
T-47D
CAMA1
MDA-MB-453
WHIM11 (PDX)
a
b
c
d
e
f
g
h
i
j
k
l
m
n
o
log2 normalized expression
ctrl
FGFR4-O/E
FGFR4-dep
ERBB2-dep
FGFR4
APOBEC3A
APOBEC3A|APOBEC3B
ns
*
* *

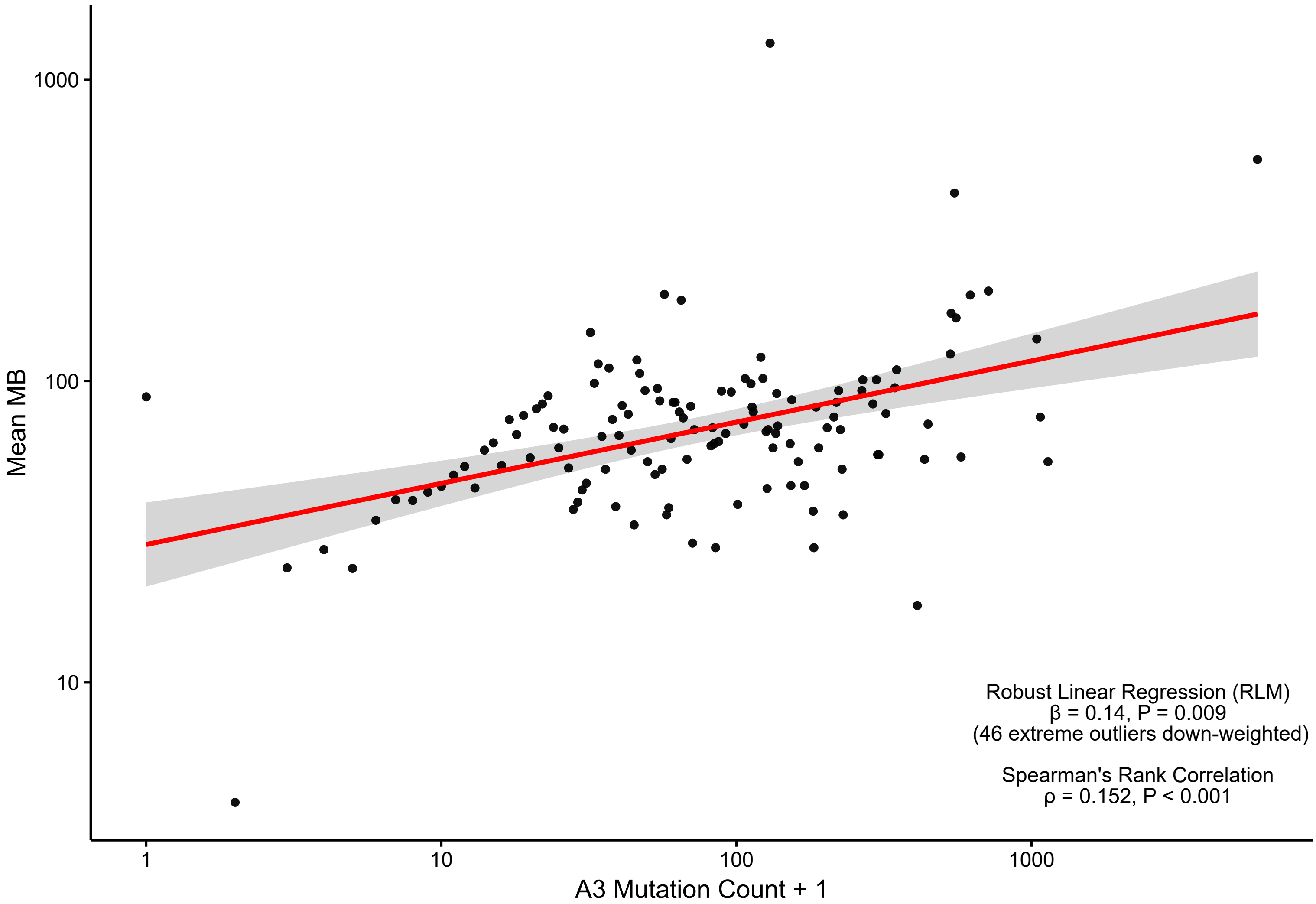
1000
100
10
Mean MB
1
10
100
1000
A3 Mutation Count + 1
Robust Linear Regression (RLM)
β = 0.14, P = 0.009
(46 extreme outliers down-weighted)
Spearman's Rank Correlation
ρ = 0.152, P < 0.001
