# Supplementary material for: FGFR4-associated APOBEC3 mutagenesis characterizes HER2-enriched subtype of human breast cancer
Source: Br J Cancer. 2026 Jul 27;135(9):1483–98. doi: 10.1038/s41416-026-03517-8 (PMC13675202; doi:10.1038/s41416-026-03517-8)
Supplement: Supplementary file 2 [file 41416_2026_3517_MOESM8_ESM.pdf]

Corresponding Author Name: Je-Yoel Cho
Manuscript Number: BJC-A3355539

SPRINGER NATURE

Reporting Summary

Springer Nature wishes to improve the reproducibility of the work that we publish. This checklist is used to ensure good reporting standards and to improve the reproducibility. Please respond completely to all questions relevant to your manuscript. For more information, please read the journal's Guide to Authors.

☑ **Check here to confirm that the following information is available in the Material & Methods section:**

- **The exact sample size (*n*)** for each experimental group/condition, given as a number, not a range
- **A description of the sample collection** allowing the reader to understand whether the samples represent technical or biological replicates (including how many animals, litters, culture, etc.)
- **A statement of how many times the experiment shown was replicated in the laboratory**
- **Definitions of statistical methods and measures**: For small sample sizes (n<5) descriptive statistics are not appropriate, instead plot individual data points
  - Very common tests, such as *t*-test, simple χ^2 tests, Wilcoxon and Mann-Whitney tests, can be unambiguously identified by name only, but more complex techniques should be described in the methods section
  - Are tests one-sided or two-sided?
  - Are there adjustments for multiple comparisons?
  - **Statistical test results**, e.g., ***P* values**
  - Definition of **'center values'** as **median or mean**;
  - Definition of **error bars as s.d. or s.e.m. or c.i.**

Please ensure that the answers to the following questions are reported in the manuscript itself. We encourage you to include a specific subsection in the methods section for statistics, reagents and animal models. Below, provide the page number or section and paragraph number.

Statistics and general methods

Statistics and general methods	**Reported in section/paragraph or page #**
1. How was the sample size chosen to ensure adequate power to detect a pre-specified effect size? (Give section/paragraph or page #)	No statistical methods were pre-employed to determine sample sizes; instead, we maximized the use of all avwailable high-quality resources to ensure the highest possible statistical power
For animal studies, include a statement about sample size estimate even if no statistical methods were used.	Regarding our in-vitro studies, we utilized all relevant breast cancer cell lines maintained within our laboratory. To ensure both biological and technical reproducibility, each experiment was performed in three independent biological
2. Describe inclusion/exclusion criteria if samples or animals were excluded from the analysis. Were the criteria pre-established? (Give section/paragraph or page #)	Inclusion and exclusion criteria were pre-established based on data integrity and standardized quality control (QC) metrics.<br><br>For computational analyses, we integrated all available
3. If a method of randomization was used to determine how samples/animals were allocated to experimental groups and processed, describe it. (Give section/paragraph or page #)	To ensure the objectivity of our findings and minimize potential selection bias, randomization was implemented across all experimental stages.<br><br>For computational analyses, no manual selection or
For animal studies, include a statement about randomization even if no randomization was used.	For in vitro studies, cell culture replicates were randomly allocated to either treatment or control groups. During the processing of assays and reagent applications, samples

4. If the investigator was blinded to the group allocation during the experiment and/or when assessing the outcome, state the extent of blinding. (Give section/paragraph or page #)	To eliminate potential observer bias, we ensured that all data assessment and experimental processes were conducted through objective and standardized methodologies.
For animal studies, include a statement about blinding even if no blinding was done.	Regarding in vitro studies, all experiments were performed in strict accordance with established protocols, with precise adherence to predetermined dosages and incubation
5. For every figure, are statistical tests justified as appropriate?	We confirmed that all statistical tests were appropriate for the data's nature and met all necessary underlying assumptions
Do the data meet the assumptions of the tests (e.g., normal distribution)?	Specifically, normality of data distribution was assessed prior to selection between parametric (e.g., t-test, ANOVA) and non-parametric tests. To ensure the validity of
Is there an estimate of variation within each group of data?	Within each figure, estimates of variation are presented as Standard Deviation (SD) to show data spread or Standard Error of the Mean (SEM) to indicate the precision of the
Is the variance similar between the groups that are being statistically compared? (Give section/paragraph or page #)	For each statistical comparison, the similarity of variance between groups was assessed (e.g., via Levene's test), or accounted for by employing appropriate statistical methods (such as Welch's t-test for unequal variances or

Reagents

	**Reported in section/paragraph or page #**
6. Report the source of antibodies (vendor and catalog number)	"N/A" (Not Applicable)
7. Identify the source of cell lines and report if they were recently authenticated (e.g., by STR profiling) and tested for mycoplasma contamination	All cell lines utilized in this study were strictly maintained at low passage numbers to preserve their original biological characteristics and were confirmed to consistently exhibit their recorded doubling times.

Animal Models

	**Reported in section/paragraph or page #**
8. Report species, strain, sex and age of animals	"N/A" (Not Applicable)
9. For experiments involving live vertebrates, include a statement of compliance with ethical regulations and identify the committee(s) approving the experiments.	"N/A" (Not Applicable)

10. We recommend consulting the ARRIVE guidelines (*PLoS Biol.* **8**(6), e1000412,2010) to ensure that other relevant aspects of animal studies are adequately reported.

Human subjects

	Reported in section/paragraph or page #
11. Identify the committee(s) approving the study protocol.	"N/A" (Not Applicable)
12. Include a statement confirming that informed consent was obtained from all subjects.	"N/A" (Not Applicable)
13. For publication of patient photos, include a statement confirming that consent to publish was obtained.	"N/A" (Not Applicable)
14. Report the clinical trial registration number (at ClinicalTrials.gov or equivalent).	"N/A" (Not Applicable)

15. For phase II and III randomized controlled trials, please refer to the CONSORT statement and submit the CONSORT checklist with your submission.

16. For tumor marker prognostic studies, we recommend that you follow the REMARK reporting guidelines.

Data deposition

	Reported in section/paragraph or page #
17. Provide accession codes for deposited data. Data deposition in a public repository is mandatory for:<br>a. Protein, DNA and RNA sequences<br>b. Macromolecular structures<br>c. Crystallographic data for small molecules<br>d. Microarray data	"N/A" (Not Applicable)<br><br>This study did not generate new primary sequencing data or macromolecular structures that require new deposition into public repositories.

Deposition is strongly recommended for many other datasets for which structured public repositories exist; more details on our data policy are available in the Guide to Authors. We encourage the provision of other source data in supplementary information or in unstructured repositories such as Figshare and Dryad. We encourage publication of Data Descriptors (see Scientific Data) to maximize data reuse.

18. If computer code was used to generate results that are central to the paper's conclusions, include a statement in the Methods section under "**Code availability**" to indicate whether and how the code can be accessed. Include version information as necessary and any restrictions on availability.	All computational analyses were conducted using standardized bioinformatic tools and R packages, with their specific names and version information explicitly documented in the Methods section.<br><br>No unique or proprietary algorithms were developed for this study; however, the custom R scripts used for data integration and visualization are available from the
