# Supplementary material for: FGFR4-associated APOBEC3 mutagenesis characterizes HER2-enriched subtype of human breast cancer
Source: Br J Cancer. 2026 Jul 27;135(9):1483–98. doi: 10.1038/s41416-026-03517-8 (PMC13675202; doi:10.1038/s41416-026-03517-8)
Supplement: Supplementary file 3 [file 41416_2026_3517_MOESM1_ESM.docx]

**Additional files**

**Additional file 1. Supplementary figures supporting the findings of this study**

**Figure S1. De novo extraction of SBS and ID mutational signatures in CMT. (**a-b) De novo signatures extracted from CMT are shown, along with their cosine similarity to decomposed human cancer signatures.

**Figure S2. Pairwise sequence similarity between human APOBEC3A-H and canine APOBEC3Z1/Z3.** (a) Sequence comparison of human and canine APOBEC3 (A3) genes. Comparisons were performed between human A3 genes and their canine counterparts, and values were normalized by the length of the canine gene sequence. (b) Structural similarity between human and canine A3 proteins. Predicted 3D structures of human APOBEC3A-H proteins and their canine counterparts (APOBEC3Z1 and APOBEC3Z3) were compared using the TM-align algorithm. Structural similarity was quantified by Root Mean Square Deviation (RMSD), where values below 2 Å indicate closer structural overlap, and by Template Modeling Score (TM-score), which ranges from 0 to 1, with higher values reflecting greater similarity in overall folding patterns. In addition, structural similarity of other cancer-related proteins including PIK3CA, PTEN, TP53, and CDH1 was also examined for comparison.

**Figure S3. Comparative analysis of sequence alignments and secondary structure between human APOBEC3 proteins and their canine orthologs.** (a-d) Predicted structural models of four protein pairs with the highest sequence similarity are shown: APOBEC3A-APOBEC3Z1, APOBEC3B-APOBEC3Z1, APOBEC3G-APOBEC3Z1, and APOBEC3H-APOBEC3Z3. Multiple sequence alignments (MSAs) were used to visualize the predicted secondary structural elements. Each protein is annotated with its gene symbol and UniProt ID. For APOBEC3B and APOBEC3G, only the sequences corresponding to the CD2 domain were included in the alignment and structural visualization. In each alignment, the first sequence in the row is numbered according to its amino acid position. Secondary structural elements such as α-helices and β-strands are displayed as squiggles and arrows, respectively. Loop regions correspond to the gaps between these structural elements. Structural elements are color-coded based on predicted Local Distance Difference Test (pLDDT) scores, with higher scores indicating greater confidence. Each amino acid residue is also color-coded according to sequence conservation: red boxes with white letters indicate strictly conserved residues (identical amino acids), and white boxes with red letters denote residues with similar physicochemical properties or high substitution compatibility. The "CMP/dCMP-type deaminase" domains are marked by bold black outlines. Additionally, the tyrosine (Y) residue located in the loop 7 region is highlighted in bold, as it is known to influence substrate specificity by promoting a preference for TpC motifs. This comparative visualization provides the structural basis for assessing whether canine APOBEC3 proteins possess the potential to induce APOBEC3-mediated mutagenesis, similar to their human counterparts.

**Figure S4. Structural confidence assessment of human and canine APOBEC3 orthologs.** (a) Predicted structural confidence (pLDDT scores) across loop regions 1, 3, and 7 in human and canine APOBEC3 proteins. Amino acid sequences were retrieved using the UniProt IDs of each protein, and five independent structural models (models 0-4) were generated using the AlphaFold server. For each protein, a single model was selected based on structural consistency and prediction confidence: APOBEC3Z1 (model 4), APOBEC3Z3 (model 2), APOBEC3A (model 0), APOBEC3B (model 1), APOBEC3G (model 1), and APOBEC3H (model 4). pLDDT scores were calculated for backbone atoms (C, N, O, and Cα), and values were aligned according to loop regions 1, 3, and 7. For human APOBEC3B and APOBEC3G, only the CD2 domain sequences were included in the analysis. Each protein is represented by a specific color in the plot: APOBEC3Z1 (red), APOBEC3Z3 (orange), APOBEC3A (yellow), APOBEC3B (green), APOBEC3G (blue), and APOBEC3H (purple). Interpretation of pLDDT values: scores >90 indicate highly reliable, accurately modeled regions; scores >70 correspond to generally confident backbone predictions; scores >50 represent low-confidence regions that should be interpreted with caution. (b-g) Predicted Aligned Error (PAE) plots for APOBEC3Z1, APOBEC3Z3, APOBEC3A, APOBEC3B, APOBEC3G, and APOBEC3H. PAE reflects the expected positional error between pairs of residues. Lower PAE values indicate higher confidence in the predicted relative spatial arrangement. Loop regions 1, 3, and 7 are marked with black dashed boxes. Overall, these confidence metrics validate the reliability of the predicted structural models, ensuring that the subsequent inter-species comparisons are based on high-quality structural data.

**Figure S5. Validation of PCR products amplified by A3Z1 and A3Z3 primers in CMT cell lines.** PCR amplification was performed using cDNA derived from five canine mammary tumor (CMT) cell lines. PCR products were separated by agarose gel electrophoresis under the following conditions: 100 V for 30 minutes. A 100 bp DNA ladder (4 μL) was loaded in the leftmost lane, and 6 μL of each sample was loaded into subsequent lanes. Bands corresponding to the expected product sizes for the A3Z1 and A3Z3 primer sets are clearly visible, confirming the specificity of the primers used for RT-qPCR.

**Figure S6. Substrates used for the in vitro APOBEC3 deaminase activity assay.** Four types of substrates were designed based on known APOBEC3 (A3) sequence and structural preferences: Linear-CCC, Hairpin-CCC, Linear-TCA, and Hairpin-TCA. A3 acts on the cytosine residues indicated by red arrows, converting them into uracil.

**Figure S7. Expression levels of APOBEC3 family genes, ERBB2, and FGFR4 in human breast cancer (HBC).** (a-f) RNA-seq expression levels of APOBEC3A (a), APOBEC3B (b), APOBEC3G (c), APOBEC3H (d), ERBB2 (e), FGFR4 (f) from TCGA-BRCA cohort. Samples were stratified by the presence or absence of A3 activity (A3+ vs. A3-) and categorized into tumor and matched normal tissue groups (A3+ tumor, n = 401; A3+ normal, n = 51; A3- tumor, n = 526; A3- normal, n = 49). Data are presented as means ± SEM. Statistical significance was determined using the two-sided Kruskal-Wallis test followed by Dunn’s multiple comparison test. *P < 0.05, **P < 0.01, ***P < 0.001; ns, not significant.

**Figure S8. Heatmap showing PAM50-based subtyping of breast cancer cell lines.** RNA-seq expression values for all genes were obtained from the DepMap portal. Subtype classification for each cell line was determined based on its PAM50 gene signature and compared with the subtype annotation provided by DepMap. Cell lines used in this study are highlighted in red. Z-score normalization was applied to expression values across genes.

**Figure S9. Validation of PCR products for ERBB2, FGFR4, APOBEC3A, and APOBEC3B primers in HBC cell lines.** PCR amplification was performed using cDNA from eight HBC cell lines, and products were separated by agarose gel electrophoresis. Bands corresponding to the expected product sizes for ERBB2, FGFR4, APOBEC3A and APOBEC3B primer sets are shown, confirming the specificity of the primers used for RT-qPCR.

**Figure S10. Microarray-based expression analysis of FGFR4 and APOBEC3A/APOBEC3B in breast cancer cell lines and patient-derived xenograft (PDX) models.** (a-o) Log₂-normalized microarray expression values for FGFR4 (a-e; NM_002011.3, probe A_23_P92754), APOBEC3A (f-j; NM_145699.2, probe A_23_P303876), and APOBEC3A|APOBEC3B (k-o; NM_145699.2|NM_004900.3, probe A_24_P66027) are shown. Each panel represents data across five breast cancer models (left to right): MCF-7 (n = 4 for control, n = 9 for O/E), T-47D (n = 4 per group), CAMA1 (n = 6 per group), MDA-MB-453 (n = 4 per group), and WHIM11 (PDX) (n = 5 for control, n = 4 per treated group). In MCF-7 and T-47D, expression levels were compared between control and FGFR4-overexpressing (O/E) groups. In CAMA1 and MDA-MB-453, comparisons were made between control and FGFR4 kinase inhibitor-treated groups. Specifically, in (h), one sample from the FGFR4-inhibitor treated group was excluded due to technical failure, resulting in (n = 5) for that specific condition. In WHIM11 (PDX), expression levels were compared among three groups: untreated control, HER2 inhibitor (Lapatinib)-treated, and FGFR4 kinase inhibitor-treated. Statistical significance was assessed by paired t test or one-way ANOVA.

**Figure S11. Quantitative dose-response relationship between APOBEC3-derived mutations and mutational burden.** To evaluate the cumulative effect of the APOBEC3 (A3) mutational process, mean mutational burden (MB) values are plotted against the gradient of A3 mutation counts on a log-log scale. The red solid line represents a linear model (lm) fit calculated to minimize the distance between data points, with the grey shaded area indicating the standard error (se) or 95% confidence interval. The strength and reliability of this relationship were further validated by additional statistical analyses; Robust Linear Regression (RLM) identified a statistically significant linear relationship where each unit increase in A3-derived mutations corresponds to an approximate 0.14 increase in total MB (β = 0.14, P = 0.009), with 46 extreme outliers down-weighted to ensure the robustness of the trend. Additionally, Spearman’s rank correlation confirmed a significant positive association between A3 activity levels and MB (rho = 0.152, P < 0.0001), reinforcing the dose-dependent nature of A3-mediated mutational accumulation.

**Additional file 2. Supplementary tables supporting the findings of this study**

**Table S1. Information of Canine mammary tumor Samples**

**Table S2. Mapping quality and coverage depth of WES datasets in Canine mammary tumor**

**Table S3. Somatic variants in coding sequences of Canine mammary tumor**

**Table S4. Statistical metrics of SigProfilerExtractor solutions for SBS96 and ID83 matrices**

**Table S5. Comparative distribution of COSMIC SBS96 and ID83 signatures across TCGA, PCAWG, and CMT cohorts**

**Table S6. Distribution of APOBEC3(+) and APOBEC3(-) oncogene hotspot mutations across HBC(A3+), HBC(A3-), and CMT cohorts**

**Table S7. Specific primers for PCR amplification and oligonucleotide sequences**

**Table S8. Differentially expressed genes (DEGs) between A3+ and A3- groups in human breast cancer (HBC).**

**Table S9. PAM50-based molecular subtype classification of breast cancer cell lines**
